# Communication strategies for improving readiness for advance care planning in dementia: A systematic review

**DOI:** 10.1177/13872877261461216

**Published:** 2026-06-26

**Authors:** Vera van der Nulft, Arianne Stoppelenburg, Liesbeth M. van Vliet, Jenny T. van der Steen, Yvette M. van der Linden

**Affiliations:** 1 Center of Expertise in Palliative Care, 4501 Leiden University Medical Center, Leiden, The Netherlands; 2 Health, Medical and Neuropsychology Unit, 4496 Leiden University, Leiden, The Netherlands; 3 Department of Public Health and Primary Care, 4501 Leiden University Medical Center, Leiden, The Netherlands; 4 Radboudumc Alzheimer Center and Department of Primary and Community Care, 6034 Radboud university medical center, Nijmegen, the Netherlands; 5 357742 Cicely Saunders Institute, King's College London, London, UK

**Keywords:** Alzheimer's disease, dementia, end of life care, psychosocial interventions, quality of health care, systematic review

## Abstract

**Background:**

Progressive cognitive decline in Alzheimer's disease and other dementias limits decision-making, emphasizing the need for timely discussions about preferred future care. However, advance care planning (ACP) is often delayed due to limited readiness among people with dementia and their family caregivers.

**Objective:**

To identify communication strategies (interventions and communication methods) improving readiness for ACP among people with dementia and their family caregivers.

**Methods:**

A mixed-methods systematic review was conducted (PROSPERO: CRD42023480187). PubMed, The Cochrane Library, Embase, PsycINFO, and CINAHL were searched in January 2024, with an update in February 2025 through expert consultation. Eligible articles included people with dementia and their family caregivers, communication strategies, and readiness for ACP. Study selection was performed independently by multiple reviewers using predefined eligibility criteria. Data were synthesized using a data-based convergent approach.

**Results:**

Of 517 identified articles, ten were included: quantitative (n = 5), mixed-methods (n = 3), and qualitative (n = 2). Eight articles described interventions, including ACP discussions, education, and self-paced, stepwise tools. Multi-component interventions combining facilitated discussions and educational components and tools showed improvements in readiness, although some outcomes were variable across subgroups or over time. Two articles explored communication methods, emphasizing the facilitator relationship and approach, timing and continuity of ACP discussions, and conversational techniques to support engagement.

**Conclusions:**

Multi-component interventions, tools, and communication methods may enhance readiness for ACP. Findings highlight the importance of tailoring ACP to individual needs and training healthcare professionals in facilitating ACP. Future research should develop a consistent framework of readiness and identify the active “ingredients” of ACP interventions.

## Introduction

Due to the progressive nature of Alzheimer's disease and other dementias and its impact on the decision-making capacity, early implementation of advance care planning (ACP) is essential.^1^ ACP for people with dementia is defined as “a process of communication about future care and treatment preferences, values, and goals with the person with dementia, family, and the health care team, preferably with ongoing conversations and documentation. This process is continued when the person with dementia becomes unable to make their own decisions”.^2^ Despite its importance, ACP discussions are frequently delayed or may not occur at all.^3–6^ This delay or absence is due to multiple barriers, including challenges in readiness among people with dementia and their family caregivers.^7,8^ A prerequisite for initiating ACP conversations is readiness, which has been defined as “preparedness for action”,^9^ “willingness to engage in ACP”,^10^ or “willingness and ability to engage in ACP”.^11^ Readiness was introduced by Fried and colleagues,^12,13^ who describe it as a process of health behavior change. This description indicates readiness is characterized by varying stages that may change over time.

Several factors contribute to the readiness to engage in ACP discussions for people with dementia and their family caregivers. Topics discussed can be considered unpleasant or emotionally distressing by some, contributing to the reluctance to initiate or engage in these conversations.^14–18^ People with dementia and their family caregivers may also first need to come to terms with the diagnosis, progressive nature and life-limiting outcome of the disease before they can start thinking about and discussing future planning.^19–21^ Therefore, establishing a trustful and supportive relationship with healthcare professionals involved in the care for people with dementia is also important for communication regarding future care decisions.^22–25^ Another factor affecting readiness stems from realistic expectations of the disease trajectory and knowledge of possible future scenarios, either due to (indirect) personal experiences with dementia or death,^14,16,26,27^ being well-informed by healthcare professionals,^15,26,28,29^ and recognizing dementia as a life-limiting illness.^30^ While readiness of people with dementia and family caregivers is crucial for engagement in ACP, healthcare professionals also face challenges in initiating and guiding these conversations. Effective communication skills are required to create a safe and supportive environment, address sensitive topics, and support informed decision-making. Healthcare professionals also play a key role in considering the appropriate timing of ACP and the receptiveness or hesitancy regarding initiation of ACP among people with dementia and their family caregivers.^31^ However, healthcare professionals often lack sufficient training on how to initiate and facilitate ACP discussions, and available guidelines and tools are often not consistently implemented, contributing to delayed engagement in ACP.^31–33^ Given that many of the factors influencing readiness are shaped within interpersonal interactions, communication can be considered a key mechanism through which readiness may be supported.

Existing approaches to support ACP in dementia care often include components aimed at strengthening or supporting communication, for example through training programs for healthcare professionals, structured ACP conversations, educational materials, and tools such as manuals or conversation aids.^3,34–36^ In this review, we refer to these approaches collectively as communication strategies, encompassing any intervention that incorporates communication-related components or specific communication method used within ACP interactions. While such strategies have shown positive effects on outcomes such as documentation of care preferences and general engagement in ACP,^3,34–36^ comparatively little attention has been paid to their impact on readiness. Readiness represents an earlier stage of the ACP process, preceding ACP actions such as documentation or engagement. As a modifiable concept and prerequisite for engagement, it may influence both the initiation and continuation of advance care planning over time, which is particularly important in dementia care where individuals may not feel ready to discuss future care despite the importance of timely engagement.

Although prior reviews have examined ACP in dementia with a focus on initiation, effectiveness, or ACP actions,^31,37–39^ none have specifically synthesized evidence on communication strategies in relation to readiness. As such, it remains unclear how different communication strategies may affect readiness as a prerequisite for engagement in ACP among people with dementia and their family caregivers and how they are applied in practice to facilitate this early stage of engagement. This systematic review aims to address this gap by providing an overview of communication strategies, including both interventions and communication methods, designed to improve readiness for ACP among people with dementia and their family caregivers.

## Methods

### Design

A systematic review of quantitative and qualitative articles was conducted. Preferred Reporting Items for Systematic Reviews and Meta-Analyses (PRISMA) was used as a reporting guideline.^40^ Qualitative and quantitative data were collected and analyzed independently and subsequently integrated to provide a comprehensive synthesis. The systematic review was registered on PROSPERO on November 6, 2023 (registration number: CRD42023480187).^41^ The study protocol of the larger research project was deemed exempt from the Medical Research Involving Human Subjects Act (WMO) by the Medical Ethics Committee (METC) of the Leiden University Medical Center.

### Search strategy

A systematic electronic search was conducted in January 2024 in the following databases: PubMed, The Cochrane Library, Embase (Ovid), PsycINFO, and CINAHL. The search terms were developed to be broad and inclusive, using a combination of MeSH terms and free-text terms for each of the four key concepts: (1) Dementia or other neurodegenerative diseases, (2) ACP, (3) Readiness, and (4) Communication. The search strategy was developed with input from a trained academic librarian and informed by search terms and strategies used in prior systematic reviews on ACP or dementia.^31,37–39^ Supplemental Table 1 provides the search terms for each database. The search strategy was pilot tested to confirm that known relevant studies were retrieved and iteratively refined through multiple rounds of discussion within the research team. During pilot testing and preliminary screening, the authors observed that eligible studies conceptualized “communication strategies” as encompassing two distinct concepts: (1) structured interventions incorporating communication-related components and (2) specific communication methods applied within ACP discussions. This distinction was used to organize the results according to the two types of communication strategies.

In addition to the multiple electronic database searches, two complementary strategies were implemented to improve comprehensiveness of the results: reference list screening of included literature reviews and expert consultation with ten experts selected from our international networks on ACP in dementia, who were asked to evaluate the list of included articles and suggest any potentially relevant missing articles that would meet the eligibility criteria. The experts were provided with a list of included articles in December 2024 and were given until February 2025 to respond.

### Eligibility criteria

Articles were included if they met the following criteria:

1. Consisted of an original empirical peer-reviewed study, including qualitative, quantitative, mixed methods, and observational studies, randomized control trials, and literature reviews;
2. Published in English;
3. Included communication strategies for ACP. The authors defined ‘communication strategies’ as any intervention incorporating communication-related components (i.e., components that structure, guide, prompt, or facilitate reflection and dialogue about ACP), such as scheduled conversations, training, education, and tools, including manuals or conversation aids, or specific communication method used within ACP discussions (e.g., conversational techniques or interactional approaches). The distinction between interventions and communication methods was made based on whether the strategy was implemented as a structured program or tool (intervention) versus a technique applied within ACP conversations (communication method);
4. Included the term ‘readiness’ or related concepts, consisting of willingness, receptiveness, acceptance, ability, attitude, preparedness, or openness to engage in ACP as an outcome. This conceptualization was developed over time through discussions between the authors, consultation with a librarian, and an exploration of relevant literature on readiness in other clinical populations.
5. Included people living with Alzheimer's disease and other dementias or cognitive impairment and/or their family caregivers.

Articles were excluded if they:

1. Did not specify any communication strategies (i.e., intervention or communication method);
2. Did not mention readiness for ACP or related concepts (as mentioned in the inclusion criteria) as an outcome.
3. Measured readiness outcomes solely through documentation, such as the completion of advance directives, rather than assessing participants’ perceptions, attitudes, or willingness to engage in ACP;
4. Only included healthcare professionals.
5. Consisted of case studies, non-published dissertations, commentaries, conference abstracts, editorial articles, study protocols, grey literature, or unpublished data.

### Study selection

Two authors (VN and AS) reviewed selected articles for eligibility based on the title and abstract using the eligibility criteria. A random ten percent of the initially identified articles (36/340) was independently screened by another author (LV), with an inter-rater reliability of 97.2% (35/36). Screening of the full texts of the remaining articles was divided between the three authors (VN, AS, and LV). Five of 65 potentially eligible articles (5/62) were independently reviewed by two authors (AS and LV), resulting in an inter-rater reliability of 80% (4/5). Reasons for exclusions were reported and in cases of disagreement, consensus was sought through discussion between three authors (VN, AS, and LV). For each literature review that was included based on the eligibility criteria, potential additional articles were identified through follow-up of the reference lists. Each of three authors (VN, AS, and LV) screened the full reference list of one literature review. Screening followed the same procedure as for the database search screening: titles and abstracts were screened using the predefined eligibility criteria. If an article was deemed potentially relevant based on the title and abstract, the full text was retrieved and assessed for eligibility. After individual screening, results were discussed among the three authors to reach consensus on final inclusion. Additional articles obtained through expert consultation were screened by one author (VN) following the same screening procedure and discussed with two other authors (AS and LV).

### Quality appraisal

Two authors (VN and AS) independently assessed risk of bias for each included study using the Mixed Methods Appraisal Tool (MMAT) version 2018.^42^ Articles were not excluded based on the results of the quality assessment; the purpose of the assessment was to evaluate the quality and possible bias. In case of disagreements in quality appraisal scores, consensus was sought through discussion between three authors (VN, AS, and LV).

### Data extraction and analysis

Data extraction was performed by one author (VN), using Microsoft Excel. Separate data extraction tables were used for qualitative and quantitative data to extract data based on the study characteristics and study results. The extraction tables were pilot tested by two authors (VN and AS), using two random included articles for each table. Extracted study details included: study design, aim, sample characteristics, study setting, methods of data collection, recruitment and sampling procedures, eligibility criteria, intervention information (if applicable), readiness definition, outcomes, and author conclusions. For quantitative articles, statistical analyses, measurement instrument(s), and outcome measures were also extracted. For qualitative articles, additional details about the qualitative methodology and the phenomenon of interest were extracted.

A data-based convergent analysis was used to integrate findings across the included articles.^43^ Definitions and conceptualizations of readiness were summarized across all included articles. Synthesis of the main findings was organized within two types of strategies: ACP interventions and specific communication methods. Quantitative data on intervention outcomes were tabulated and qualitized (i.e., converted from statistical results into descriptive patterns of intervention characteristics and effects). Qualitative data on interventions were integrated with the qualitized quantitative results. For communication methods, key themes or categories related to ACP readiness were extracted from qualitative articles, tabulated, and categorized into self-developed themes. First, data were synthesized separately for each type of strategy, using tabulation and textual descriptions to provide a structured narrative of the findings. Next, integration was achieved by linking the two types of communication strategies. The analysis was carried out by one author (VN) and reviewed by two additional authors (AS and LV) to ensure consensus on theme development and data interpretation. Only data regarding ACP readiness were reported in the review.

## Results

### Search and expert consultation results

**Figure 1.**
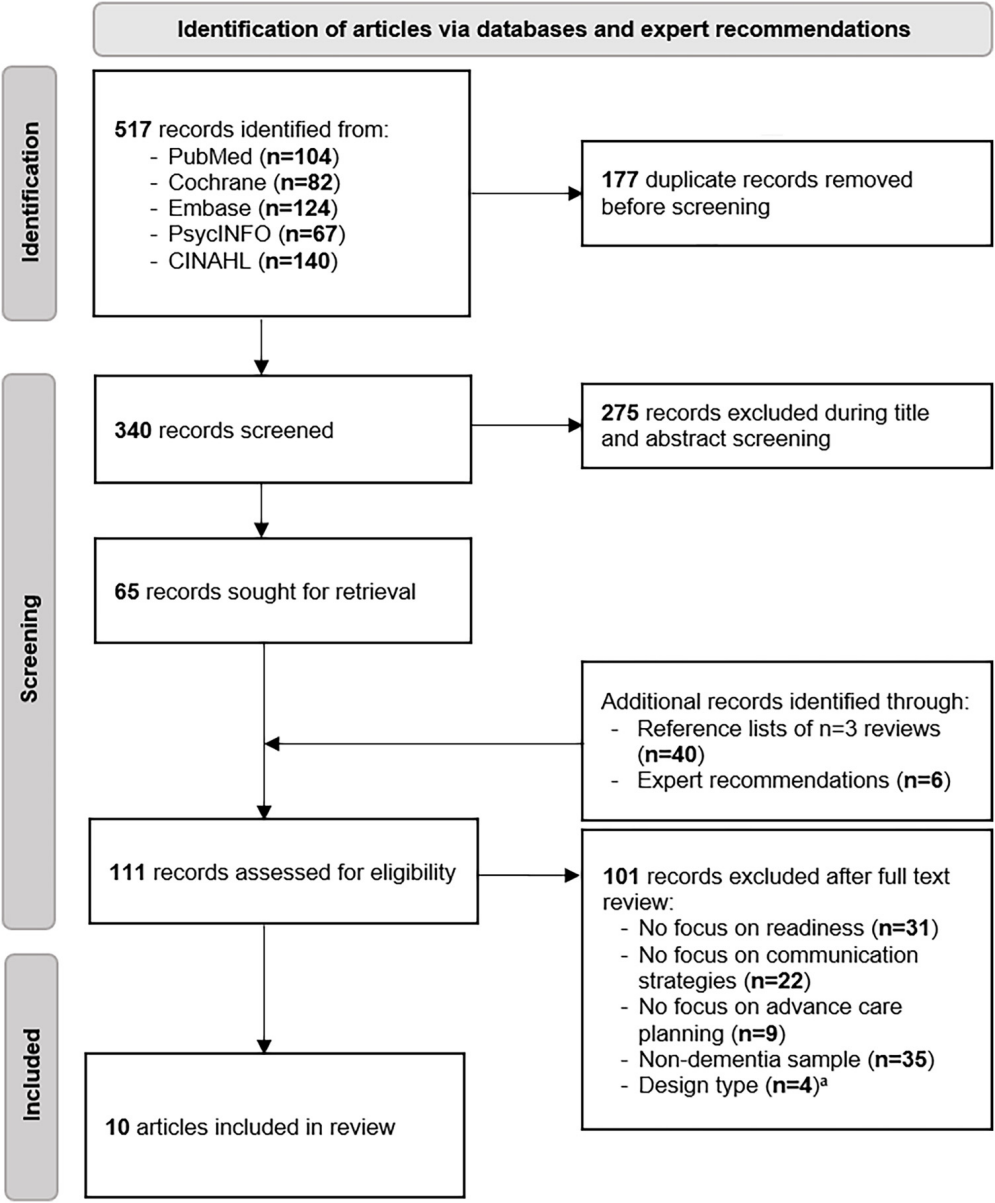
PRISMA diagram of identification of relevant articles.

^a^Three records were overview articles and one was a handbook, which were excluded as they did not present original empirical data.

Details of the search results and exclusion of articles can be found in Figure 1. With the systematic electronic search, 517 articles were identified. After de-duplication, 340 articles remained. The authors excluded 275 articles based on title and abstract. All ten experts contacted to verify the list of included articles responded. Eight experts reviewed the list, with four of them identifying additional articles and four confirming that the list appeared comprehensive. Two experts reported being unfamiliar with the most current literature on ACP in dementia. After the addition of articles identified by the experts’ input (n = 5) and a manual search of reference lists of three included literature reviews (n = 40), the full-text screening was completed for 107 articles. In total, ten articles met the eligibility criteria for inclusion.^44–53^

### Study characteristics

Table 1 shows that of ten included articles, five had a quantitative design,^45,46,51–53^ three were mixed-methods,^44,47,50^ and two were qualitative.^48,49^ The studies were conducted in Belgium,^48,50^ Taiwan,^45^ Singapore,^44^ Canada,^47,53^ China,^52^ and the United States of America.^46,49,51^ An overview of the full-text screening is presented in Supplemental Table 3, and data extraction sheets of all included articles are provided in Supplemental Table 4 and 5.

**Table 1.** Study characteristics and communication strategies for readiness for advance care planning (ACP) in dementia (N = 10).

Authors (year)	Country	Study design	Sample characteristics	Care setting	Readiness definition used	Communication strategy description	Measurement instrument	Outcomes of readiness for ACP
**Interventions to improve readiness for ACP**								
Huang et al. (2020)^45^	Taiwan	Quantitative study (pre- and post-intervention questionnaires)	N = 40 dyads of people with mild dementia (n = 40) and family caregivers (n = 38)	Hospital and community centers	“ACP attitude”	The first part of the intervention provided dyads of people with dementia and family caregivers with a manual regarding ACP (‘Be My Own Master: Talk about Advance Care Planning for Dementia’). A senior nurse trained in ACP explained the contents of the manual. The manual included descriptions about the symptoms of end-stage dementia and the common end-of-life life-sustaining medical treatments (e.g., CPR, machine ventilation, tube feeding, intravenous infusion, and antibiotics), provided details about the benefits and risks of the treatments. Additionally, the manual described the process of establishing ACP and the regulations involved. The second part involved a nurse-facilitated open discussion between patients and family caregivers, where dyads were encouraged to communicate their thoughts and expectations about end-of-life care, and to describe any uncertainties about the process. The intervention lasted approximately 60 min.	A 17-item Advance Care Planning Attitude scale, modified from a scale measuring positive and negative attitudes towards Taiwan's Hospice-Palliative Care Act,^54^ scored on a 5-point Likert scale.	People with dementia showed no significant changes in ACP attitude. Family caregivers showed decreased negative attitude, but no significant change in positive attitude. People with dementia: – Positive attitude: Pre-intervention (Mean=33.00, SD = 6.09) versus post-intervention (Mean=34.98, SD = 7.01), *p*=0.126 (95% CI: −4.52 to 0.57, effect size=0.3). – Negative attitude: Pre-intervention (Mean=23.03, SD = 4.02) versus post-intervention (Mean=22.93, SD = 3.06), *p*=0.908 (95% CI: −1.63 to 1.83, effect size=-0.02). Family caregivers: – Positive attitude: Pre-intervention (Mean=40.08, SD = 4.47) versus post-intervention (Mean=39.08, SD = 4.59), *p*=0.149 (95% CI: -0.37 to 2.35, effect size=-0.2). – Negative attitude: Pre-intervention (Mean=24.27, SD = 4.12) versus post-intervention (Mean=20.65, SD = 5.26), *p*=0.001 (95% CI: 1.65 to 5.58, effect size=-0.7).
Ali et al. (2021)^44^	Singapore	(Parallel) mixed-method study (Pre- and post-intervention questionnaires and post-intervention interviews)^a^	N = 20 dyads of people with mild dementia and family caregivers	Hospital	“Willingness” and “readiness for ACP”	A trained ACP facilitator (a dementia nurse) conducted a scheduled ACP counseling session with dyads of people with dementia and family caregivers after diagnosis. The session consisted of educating dyads of people with dementia and family caregivers on ACP, exploring fears and concerns in initiating ACP, and discussions on preferred future care plans. The intervention lasted approximately 60 min. A short debriefing session was arranged to address any concerns or queries that might have surfaced during the counseling session and follow-up plans were carried out if required.	‘Would like further discussion’-item of a self-developed questionnaire on perceptions of ACP, based on a study by Fried and colleagues.^55^	Willingness to have further ACP discussions increased for people with dementia and decreased for family caregivers: – People with dementia: Pre-intervention (55%, 11/20) versus post-intervention (68%, 13/19). – Family caregivers: Pre-intervention (85%, 17/20) versus post-intervention (79%, 15/19). Differences in willingness between people with dementia and family caregivers were not statistically significant, neither pre-intervention (*p*=0.082) nor post-intervention (*p*=0.714). Changes in willingness from pre- to post-intervention within each group were not statistically tested.
Sussman et al. (2022)^47^	Canada	(Sequential) mixed-methods study^b^	N = 18 (n = 10 people with dementia and n = 8 family caregivers)	Community center and home care	“Readiness for ACP engagement”	Your Conversation Starter Kit workbook: Developed by the Institute for Healthcare Improvement as a part of a public engagement initiative to improve ACP awareness and uptake for all adults. The workbook is an interactive 12-page tool informed by a staged model of ACP engagement. Workbooks were given by the researchers. The four steps included in the workbook were designed to move people through a process of personal reflection and communication. Each section contains open-ended questions. The second section also contained ranked choice questions. The first two steps (Get Ready & Get Set) promoted reflection on how to engage with a family member/caregiver in ACP discussions through tips, prompts, and questions that focus on values, concerns, and preferences for future care. The last two steps (Go & Keep Going) directed users towards discussions with family/caregivers.	No measurement instrument was used (outcomes are based on focus groups).	The workbook was reported to support initial and ongoing ACP engagement for people with dementia and family caregivers: – *Initiation of ACP:* Participants who had no prior engagement with ACP felt the workbook could be used to “open the conversation” because the content “gives ideas” and “good prompts” for what to think about and discuss. – *Stepwise preference*: The direction offered by the workbook was viewed as useful for initial ACP engagement because people “don’t always know what to think about because people have not faced it yet”. – *Maintenance of ACP*: Participants who had previously engaged in ACP also saw utility in the workbook because they felt it could reinforce, maintain, or elaborate on former future care discussions and reflections.
Vellani et al. (2022)^53^	Canada	Quantitative study (non-randomized)	N = 20 dyads of people with mild dementia and family caregivers	Outpatient geriatric clinics	“Readiness” and “ACP engagement”	The Voice Your Values (VYV) intervention: An intervention based on empirical data and the theoretical underpinnings of the Representational Approach to Patient Education,^56–58^ and the Transtheoretical Model of Stages of Change.^59^ The process of delivering the intervention was guided by a selection of recommendations by Piers and colleagues: (1) timing to initiate ACP, (2) capacity evaluation, (3) carrying out ACP discussions, (4) role of those close to the person with dementia, and (5) documentation of wishes.^60^ The intervention consisted of two sessions using videoconferencing: (1) The first session consisted of gathering information about the participant's understanding of dementia, the desire to learn about dementia and ACP, life goals, fears and worries related to the dementia diagnosis. (2) The second session consisted of a summary from the first session, tailored education about dementia and ACP, addressing in-the-moment questions by the participants, coaching the participants to identify vales and wishes for future healthcare (related to terminal and vegetative states), and documentation of values and wishes.	Advance Care Planning Engagement Survey (only the readiness and self-efficacy subscales).^61,62^	People with dementia showed a significant increase in ACP readiness and self-efficacy after the intervention, with a large effect size. No readiness were reported for family caregivers. People with dementia: ACP Engagement Survey: – Baseline: Mean=3.2 (SD = 0.6). – Follow-up: Mean=3.7 (SD = 0.8). – Change: 0.5 (95% CI: 0.3–0.7), p = 0.00014, effect size=1.1. Categorization of Stage of Behavior: – Pre-contemplation at baseline: 55% (n = 11) – Pre-contemplation at follow-up: 40% (n = 8) – Change: 15% progressed to higher stages, p = 0.32
Yeung et al. (2023)^52^	China	Quantitative study (non-randomized)	N = 36 dyads of people with dementia and family caregivers	Community centers	“Readiness for ACP” and “readiness for ACP engagement”	Have a Say (HAS) program: A culturally appropriate dyadic ACP intervention for Chinese people with early-stage dementia, underpinned by the Bandura's self-efficacy model. It encompasses four sources of self-efficacy of the model: vicarious experience, verbal persuasion, emotional arousal, and performance accomplishment. The program consisted of three weeks of sessions aimed at facilitating ACP for people with dementia: (1) In week 1, participants engaged in a nurse-led group session with a micro-movie, video clips, and group discussions to increase understanding of dementia, ACP, and future care options. They also reflected on their views on future health decline and end-of-life care preferences. (2) Week 2 focused on facilitator-led dyadic sessions, where participants explored their values and preferences for future care using a booklet. Topics included personal values, place of care, daily care arrangements, and feeding options. (3) In week 3, the focus shifted to decision making, where dyads made decisions on life-sustaining treatments, legal and financial matters, surrogate decision-makers, funeral arrangements, and any other unfulfilled wishes, all of which were documented in the ACP booklet.	Advance Care Planning Engagement Survey (Chinese version).^61–63^	Dyads of people with dementia and family caregivers in the intervention group showed a significantly greater improvement in readiness for ACP compared to the control group immediately post-intervention, but there were no significant differences between the groups at 1-month or 3-month post-intervention: – Baseline: Intervention (Mean=1.74, SD = 1.08) versus Control (Mean=1.06, SD = 0.13), group effect *p*=0.007 (95% CI: 0.19 to 1.16). – Immediately post-intervention: Intervention (Mean=2.49, SD = 1.00) versus Control (Mean=1.20, SD = 0.28), time effect *p*=0.070 (95% CI: -0.01 to 0.28), group*time interaction *p*=0.006 (95% CI: 0.15 to 0.92, effect size=1.76). – 1-month post-intervention: Intervention (Mean=2.13, SD = 0.99) versus Control (Mean=1.41, SD = 0.50), time effect *p*=0.006 (95% CI: 0.10 to 0.57), group*time interaction *p*=0.925 (95% CI: -0.52 to 0.47, effect size=0.92). – 3-month post-intervention: Intervention (Mean=2.24, SD = 1.17) versus Control (Mean=1.18, SD = 0.34), time effect *p*=0.538 (95% CI: -0.14 to 0.27), group*time interaction *p*=0.088 (95% CI: -0.06 to 0.82, effect size=1.23).
Ouchi et al. (2024)^46^	USA	Quantitative study (pre- and post-intervention questionnaires)	N = 26 dyads of people with mild cognitive impairment or dementia and family caregivers	Hospital	“ACP engagement” and “ready”	ED GOAL: A short, scripted, motivational interview-based discussion, which prompted people with dementia and family caregivers to articulate their values and preferences, and communicate them in ACP conversations with their healthcare professionals. The discussions were conducted by trained research nurses. Training consisted of one hour of learning about motivational interviewing and serious illness communication skills, followed by four hours of communication training with trained actors.	Advance Care Planning Engagement Survey.^61,62^	There was no significant change in overall ACP engagement of people with cognitive impairment or dementia and family caregivers: – Readiness to sign papers for a decision-maker: Pre-intervention (Mean=4.7, SD = 1.0) versus post-intervention (Mean=5.0, SD = 0.2), *p*=0.141. – Readiness to talk about future care and treatment to decision-maker: Pre-intervention (Mean=4.2, SD = 1.3) versus post-intervention (Mean=4.6, SD = 0.8), *p*=0.872. – Readiness to talk about future care and treatment to healthcare professional: Pre-intervention (Mean=3.4, SD = 1.5) versus post-intervention (Mean=4.2, SD = 1.1), *p*=0.364. – Readiness to sign papers for future care and treatment wishes: Pre-intervention (Mean=3.8, SD = 1.5) versus post-intervention (Mean=4.1, SD = 1.5), *p*=0.336.
Wolff et al. (2024)^51^	USA	Quantitative study (randomized controlled trial)	N = 273 dyads of people with mild, moderate and severe cognitive impairment and family caregivers	Primary care	“Readiness to engage in ACP”	Sharing Health Care Wishes in Primary Care (SHARE): Consisted of standard care, with the addition of the following: (1) A letter from the primary care practice, introducing a new initiative to improve communication with information about planning an appointment, the patient portal, and ACP. (2) Ongoing access to a trained facilitator (nurse, social worker or lay health worker) who leads the ACP discussions, based on the Respecting Choices conversation guide ^64,65^. (3) Planning an appointment with the people with cognitive impairment and family caregivers to discuss the role of family and stimulate discussions about goals of care and ACP. (4) Facilitated registration to an electronic health record (i.e., patient portal) for people with cognitive impairment and family caregivers.	Advance Care Planning Engagement Survey.^61,62^	People with cognitive impairment in the intervention group showed higher readiness to engage in ACP than the control group at baseline and at 12 months, with no significant difference at 6 months. Family caregivers in the intervention group showed greater readiness at baseline and at 6 months, but the effect was not sustained at 12 months. People with cognitive impairment: – Baseline: Intervention (Mean=16.29, SD = 4.2) versus control (Mean=14.41, SD = 5.2), *p*=0.014. – 6 months post-intervention: Intervention (Mean=17.25, SD = 3.5) versus control (Mean = 16.21, SD = 4.7), *p*=0.459 (adjusted). – 12 months post-intervention: Intervention (Mean=18.00, SD = 3.8) versus control (Mean=15.52, SD = 4.5), *p*=0.042 (adjusted). Family caregivers: – Baseline: Intervention (Mean=24.58, SD = 6.3) versus control (Mean=23.00, SD = 7.0), *p*=0.051. – 6 months post-intervention: Intervention (Mean=27.01, SD = 4.2) versus control (Mean=24.82, SD = 6.9), *p*=0.010 (adjusted). – 12 months post-intervention: Intervention (Mean=25.96, SD = 6.4) versus control (Mean=25.19, SD = 6.7), *p*=0.435 (adjusted).
Dupont et al. (2024)^50^	Belgium	(Convergent parallel) mixed-methods study^a^	N = 52 (n = 21 people with dementia and n = 31 family caregivers)	Community centers and hospital	“ACP readiness” and “readiness for ACP engagement”	An ACP support website, with the aim to inform people with dementia and their family caregivers about ACP and to facilitate their engagement in the ACP process, specifically by enhancing their knowledge, attitudes, self-efficacy, and skills. To address these aims, the authors drew upon various theories, such as protection motivation theory and social cognitive theory. These theories guided the selection of behavior change methods aimed at achieving the website's goals, which were then translated into the website's content. The website contained the following: (1) Information about ACP and its legal frameworks, why ACP is important, and reasons to discuss ACP with the healthcare professional (supported by videos). The website allowed users to navigate according to their preferences, allowing a non-linear approach (i.e., people with dementia and their family caregivers can use the website based on their needs and readiness levels); (2) Support for communication by tips and videos on how to start talking about ACP and how to introduce the topic with the healthcare professional; (3) Information about the documentation of ACP and referral to healthcare professionals; (4) Two interactive communication support tools to support reflection and communication about what matters most and social aspects of future care and treatment, and end-of-life preferences: a card game with preformulated statements, based on the Go Wish card game,^66^ and a fill-in tool with open-end questions about “what matters to you”; (5) Several functionalities to support accessibility of the information (e.g., text-to-speech, enlarge-the-text and contrast options).	Advance Care Planning Engagement Survey (Dutch version).^61,62,67^	More people with dementia expressed ACP readiness after the intervention compared to baseline. More family caregivers showed readiness at baseline than people with dementia and more expressed readiness for ACP following the intervention compared to baseline. People with dementia: – Wants to or documented a proxy decision-maker: Pre-intervention 67% versus post-intervention 86%. – Wants to or discussed future care and treatment possibilities with family caregiver: pre-intervention 57% versus post-intervention 67%. – Wants to or discussed future care and treatment possibilities with healthcare professional: Pre-intervention 57% versus post-intervention 71%. – Wants to or documented possibilities for future care and treatment: Pre-intervention 38% versus post-intervention 71%. Family caregivers: – Wants to or documented a proxy decision-maker: Pre-intervention 81% versus post-intervention 93%. – Wants to or discussed future care and treatment possibilities with person with dementia: pre-intervention 61% versus post-intervention 87%. – Want to or discussed future care and treatment possibilities with healthcare professional: Pre-intervention 68% versus post-intervention 77%. – Wants to or documented possibilities for future care and treatment: Pre-intervention 68% versus post-intervention 81%. Changes in ACP readiness from pre- to post-intervention within each group were not statistically tested.
**Communication methods to improve readiness for ACP**								
Van Rickstal et al. (2019)^48^	Belgium	Qualitative study (focus groups)	N = 15 family caregivers of people with early-onset dementia	Community center and hospital	“Engagement in ACP” and “ACP engagement”	The study describes family caregivers’ preferences for how to ideally engage them and their loved one with dementia in ACP.	No measurement instrument was used (outcomes are based on semi-structured interviews).	Family caregivers of people with early-onset dementia reported multiple preferences for ACP: – *Initiator role*: The ideal person to initiate ACP would be a third party, mainly physicians. One person felt a peer, or a ‘buddy’, would be best suited to introduce ACP. The physicians’ attitudes also plays a role; there was a preference for honest and empathic physicians over those who did not seem to see their patients as a person. – *Timing*: The ideal timing for initiating ACP was perceived slightly different by several family caregivers. The majority felt that initiation should take place soon after diagnosis. Others noted the necessity of allowing the patient time to process the diagnosis. – *Patient-professional relationship*: Establishing a trusting relationship between patient and physician prior to ACP is of importance. – *Discussion over time*: Repeatedly discussing preferences instead of just once was preferred. Advance directives were seen as a potential means to start a broader conversation about future care to emphasize patients’ remaining abilities, rather than emphasize their acquired limitations. – *Individuality*: Several participants mentioned an ‘individual approach’ is of great importance. – *Education*: Participants expressed the need to educate people on the existence of ACP and to explain it in a simple manner. It was also recommended possible care options be clarified.
Abshire Saylor et al. (2024)^49^	USA	Qualitative study (thematic conversation analysis)	N = 88 dyads of older adults with cognitive impairment and family caregivers	Primary care	“Engagement in ACP”	Sharing Health Care Wishes in Primary Care (SHARE): See description of article of Wolff and colleagues (2024).^51^	No measurement instrument was used (outcomes are based on analysis of ACP discussion transcripts).	Structured questions about values and strong ACP communication strategies elicit engagement from older adults with dementia: – *Values elicitation*: Clarifying the purpose of ACP and asking what constitutes “a good day” or good quality of life (as part of the Respecting Choices conversation guide) were successful strategies in engaging people with cognitive impairment in ACP discussions. Values-elicitation questions regarding cultural or spiritual values allowed some participants to share information about their faith and the communities with whom they identified. In one case, having a trained facilitator with cultural congruence to the participants helped elicit engagement and communication. – *Repetition and mirroring*: The trained facilitators successfully used communication strategies such as repetition and mirroring to confirm their understanding of the values of the participant. This was particularly important when family caregivers dominated during the ACP conversation. These communication strategies allowed older adults with dementia to affirm their wishes.

a Only the quantitative results of these articles were reported, as the qualitative findings did not consider readiness as an outcome.

b Only the qualitative results of this article were reported, as the quantitative findings did not consider readiness as an outcome.

Effect sizes, confidence intervals, and statistical significance were reported where available.

### Quality appraisal

The full quality appraisal of all included articles and the rationale for the scores are provided in Supplemental Table 2. Most included articles were rated as good quality using the Mixed Method Appraisal Tool.^42^ One quantitative study received a low score (i.e., 2/5) due to differences between the control and intervention groups, incompleteness of the data, and not accounting for confounding factors.^52^

### Readiness definition

The included articles demonstrated various terms to describe the concept of readiness for ACP, as presented in Table 1. Most articles used terms closely aligned with “readiness,” although others framed it more broadly in relation to ACP engagement, willingness, or attitudes. While wording varied, two main conceptualizations emerged: (1) readiness as measured by the ACP Engagement Survey, which captures multiple domains of ACP behavior change (knowledge, contemplation, self-efficacy, and explicit readiness for specific ACP actions), and (2) attitudinal or emotional preparedness, reflected in willingness items or attitudinal scales.^44,45^ Only one article explicitly defined readiness as the pre-contemplation stage of behavior change,^44^ while the others provided no formal definition.

### Communication strategies for readiness for ACP

Table 1 outlines interventions and communication methods and outcomes of the included articles (N = 10). Eight articles included interventions,^44–47,50–53^ and two discussed communication methods.^48,49^

*Interventions to improve readiness for ACP interventions.* Various interventions aimed at enhancing readiness for ACP among people with dementia and their family caregivers were described. Interventions of the included articles shared a common goal of improving understanding, engagement, and communication about future care decisions. Overall, interventions primarily targeted dyads consisting of the person with dementia and a family caregiver, with the goal of supporting reflection on values and preferences for future care.^44–46,51–53^ These discussions were all facilitated by healthcare professionals who received specific training as part of the intervention. Across studies, interventions incorporated educational components to inform participating people with dementia and family caregivers about ACP, care options, and legal frameworks. The mode of delivery of education varied across interventions: education was provided through written materials,^45,47,50^ videos,^50,52^ or provided by trained healthcare professionals during ACP discussions.^44,45,51–53^ Two interventions consisted of self-paced and stepwise tools, including a website and a workbook,^47,50^ designed to allow participants to engage in ACP at their own pace and according to their needs. Increases in readiness for ACP were reported among interventions that combined facilitated discussions with educational components or included tools.^44,45,47,50–53^ Effects on readiness for ACP were absent for a single-component intervention that relied solely on a facilitated conversation.^46^ For some interventions, outcomes varied between people with dementia and family caregivers, with differences in the direction of change in outcomes across these subgroups observed within the same intervention.^44,45,51^ However, no consistent pattern emerged across studies regarding these subgroup differences. In articles with repeated follow-up measurements, interventions improved readiness immediately post-intervention. However, these effects were not sustained at short-term follow-up (1–3 months).^52^ At longer-term follow-up (6–12 months), findings were mixed and differed between subgroups, with some sustained effects for people with cognitive impairment at 12 months, while effects among family caregivers were not maintained over time.^51^ Furthermore, no consistent patterns emerged with respect to care setting or level of cognitive impairment. Interventions were conducted across diverse contexts, including hospital, community, outpatient, and primary care settings, and involved people with mild to more advanced cognitive impairment.

*Communication methods to improve readiness for ACP.* Two articles explored specific communication methods (i.e., approaches or techniques to be used during ACP discussions) to support readiness for ACP among people with dementia and family caregivers.^48,49^ Three key themes emerged. First, facilitator relationship and approach: readiness can be supported when ACP discussions are led by a healthcare professional or another independent person (e.g., a buddy or peer). Preferably, a facilitator with whom the person with dementia or family caregiver has a trustful relationship and who is honest, empathetic, and treats the person with dementia as an individual, respecting their personal values and needs.^48^ Second, timing and continuity of discussions: readiness may increase when the timing of ACP discussions is tailored to individual needs and revisited over time, allowing the person to gradually engage in the ACP process.^48^ Third, conversational techniques to support engagement: readiness may be improved by approaches that help people with dementia to communicate their preferences, including eliciting personal values (e.g., asking simple questions about quality of life and cultural or spiritual considerations), repeating or mirroring discussion points, and providing clear explanations about the purpose of ACP.^49^

*Linking interventions and communication methods.* Across studies, communication methods (e.g., facilitator relationship and approach, timing and continuity of discussions, and conversational techniques) were embedded within multi-component interventions and self-paced tools. All interventions including facilitated discussions emphasized the facilitator relationship and approach: trained healthcare professionals led discussions with dyads or group sessions and often provided ongoing support through multiple follow-up sessions.^44–46,51–53^ Timing and continuity were addressed in multi-component interventions that included stepwise sessions, scheduled follow-ups, or repeated facilitator contact, allowing gradual engagement in ACP discussions.^44,45,51–53^ Conversational techniques to support engagement, mainly eliciting personal values and providing clear explanations about the purpose of ACP were described in some interventions,^46,52,53^ whereas techniques such as mirroring or repeating discussion points were not reported.

## Discussion

### Main findings of this review

To our knowledge, this is the first systematic review to provide an overview of ACP communication strategies to improve readiness for ACP among people with dementia and their family caregivers. However, given the small number of articles included in this review, their variable quality, and incomplete reporting of quantitative outcomes in some articles, recommendations for implementation are made with caution.

The findings suggest that interventions combining facilitated discussions with educational components may have the potential to increase readiness among people with dementia and family.^44,45,51–53^ This aligns with the framework of complex interventions, which incorporate multiple interacting components and engage different stakeholders,^54,55^ indicating that multi-component interventions may be well-suited to address the multifaceted nature of ACP readiness. However, in some multi-component interventions included in this review, initial increases in readiness diminished following the intervention,^51,52^ suggesting that ongoing support or reinforcement may be helpful to sustain engagement in ACP among people with dementia and their family caregivers. Incorporating booster strategies into intervention designs, such as scheduled follow-up conversations, brief refresher sessions, or prompts delivered through digital platforms, may be important to support continued readiness for ACP. Future research should also include longer follow-up periods to better assess the sustainability of intervention effects, as well as evaluate booster strategies to support ongoing readiness for ACP. Some outcomes also differed between people with dementia and family caregivers within interventions,^44,45,51^ although no consistent pattern in the direction of these differences was observed across studies. Given the distinct roles and perspectives of people with dementia and family caregivers within ACP, future research could further examine and explore differences between these groups in their responses to interventions. The findings also indicate that the use of self-paced, stepwise tools is promising for improving readiness for ACP.^47,50^ Although it does not consist of multiple components, these tools provide structured, stepwise guidance and allow participants to engage at their own pace, supporting reflection and articulation of values and preferences in a manner tailored to individual needs.

Findings on communication methods may further suggest that readiness is not only influenced by exposure to structured multi-component interventions or tools but also by communication methods, including facilitator relationship and approach, timing and continuity of ACP discussions, and conversational techniques to support engagement. The way facilitators interact with people with dementia and family caregivers may influence readiness, as honesty, empathy, and respect can reinforce the trustful relationship and help to engage in ACP,^48^ consistent with prior research.^22–25^ The identified communication methods identified in this review could help inform how healthcare professionals may engage people with dementia and their family caregivers in ACP discussions. These methods also highlight the potential value of training healthcare professionals in specific communication methods that may affect readiness for ACP. Timing and continuity also seem to be critical for readiness, as individuals were shown to differ in their preference for when to initiate ACP discussions and revisit them over time.^48^ This aligns with previous research indicating that some people with dementia and family caregivers may need time to process the diagnosis before they feel ready to initiate ACP,^19–21^ and that healthcare professionals play a crucial role in estimating the right timing for initiating ACP.^31^ Readiness may also be supported by conversational techniques that help people with dementia to communicate their preferences during facilitated ACP discussions, such as asking simple questions about personal values, repeating preferences of the person with dementia, and mirroring them.^49^

### Strengths and limitations

Strengths of this review are the inclusion of a variety of study designs and consideration of multiple definitions of “readiness,” which allows for a more comprehensive and nuanced understanding of the topic. By including both interventions and perspectives on communication methods, the review expands beyond a sole focus on intervention effectiveness, offering insights into the preferences and needs of people with dementia and their family caregivers. Another strength of this review is the comprehensive search strategy, which included multiple major databases and supplementary search methods such as reference list screening and expert consultation to maximize the identification of relevant studies.

Several limitations should also be acknowledged. Only articles published in English were included, which may have introduced language bias and led to the exclusion of relevant studies conducted in non-English-speaking or ethnically diverse populations. In some populations, dementia stigma and the lack of a norm for disclosing a diagnosis can limit discussions and influence readiness for ACP. Similarly, the search strategy focused exclusively on peer-reviewed journal articles, which could have limited the inclusion of relevant studies in grey literature. These factors may have affected the comprehensiveness of the results. Moreover, despite efforts to capture a broad range of terminology related to readiness, variation and ambiguity in the definitions and conceptualizations of “readiness” and related concepts (e.g., “attitude,” “willingness,” “engagement”) required the research team to make decisions based on intersubjectivity during study selection and synthesis. For example, “attitude” was defined by the research team as reflecting one's openness to ACP, “willingness” as reflecting emotional readiness, and “engagement” as a manifestation of readiness through active participation. Consequently, the lack of explicit definitions of readiness or related concepts in most included articles and the need for interpretation may have influenced the comprehensiveness and consistency of the review's findings.

### Conceptualization, measurement, and role of readiness in ACP research

This review presents several implications for future research. The observed variations in terminology across the included articles reflect the diverse conceptualization of readiness for ACP. While readiness for ACP has been more clearly defined for the general older population,^10–13^ there is still no single universally accepted definition. In addition, although some validated measurement instruments were used across various interventions found in this review, there is still considerable variation in how readiness for ACP is measured. This variation in terminology and measurement of readiness, combined with a lack of explicit conceptualizations of readiness in the majority of the included articles, limits comparability of findings and may influence interpretation of outcomes. Future research should focus on developing a universal definition of readiness that applies across different populations and using standardized measurement instruments. This would enhance the comparability across studies, improve the applicability of findings, and support more effective interventions.

The extent to which findings across studies can be meaningfully synthesized in relation to how readiness is associated with ACP engagement is also limited. While increased readiness does not necessarily translate into subsequent ACP engagement, most communication strategies included in this review are part of the ACP process, either as components of structured interventions (e.g., facilitated discussions) or as communication methods applied during ACP interactions. This indicates that readiness may not only function as a prerequisite to ACP engagement but may also emerge as a process outcome of the ACP process itself, in line with previous literature.^56,57^ This suggests that there is a more complex and bidirectional relationship between readiness and communication, highlighting the need for future research to further examine how this relationship develops over time in the context of ACP. Exploring the active “ingredients” of intervention components that contribute to readiness for ACP, for example, through realist synthesis or process evaluations, would further strengthen the evidence base.

While interest in readiness for ACP of people with dementia appears to be growing, as indicated by more consistent use of similar measurement instruments and an increase in research about readiness in recent years, many evaluations of ACP interventions do not assess their impact on readiness. Various ACP interventions have been developed for people with dementia or cognitive impairment, including we DECide,^58^ ENACT,^59^ PriDem,^60^ ACP-ED,^61^ the SHARE program,^62^ the LEAD guide,^63^ SHARING Choices,^64^ SPIRIT,^65^ mySupport,^66^ and others.^67–76^ These interventions are well-received and associated with improved communication, reduced uncertainty, and better decision-making. However, despite the importance of readiness, research evaluating interventions has primarily focused on other ACP outcomes, particularly in dementia care. In contrast, readiness for ACP is more extensively studied in other clinical domains, such as oncology and cardiology, as well as within the general older adult population.^77–81^ In dementia, where cognitive decline progressively limits decision-making and there is a need for early involvement of family caregivers, timely ACP is crucial, underscoring the need to prioritize the readiness of people with dementia and family caregivers. Future research should therefore focus on evaluating the impact of existing and emerging ACP interventions on readiness in dementia care.

### Conclusion

This review examined communication strategies, consisting of interventions and communication methods, for improving readiness for ACP among people with Alzheimer's disease and other dementias and their family caregivers. Multi-component interventions combining facilitated ACP discussions and educational components and self-paced, stepwise tools may have the potential to improve readiness. Communication methods, including facilitator relationship and approach, timing and continuity of ACP discussions, and conversational techniques, may also support readiness for ACP. The findings highlight the importance of tailoring ACP to the unique needs of the person with dementia and their family caregivers and training healthcare professionals in how they can facilitate ACP. Future research should focus on developing a consistent framework of readiness and exploring the active ingredients of ACP interventions to better understand which components most effectively support the readiness of people with dementia and their family caregivers.

## Supplemental Material

sj-docx-1-alz-10.1177_13872877261461216 - Supplemental material for Communication strategies for improving readiness for advance care planning in dementia: A systematic review Supplemental material, sj-docx-1-alz-10.1177_13872877261461216 for Communication strategies for improving readiness for advance care planning in dementia: A systematic review by Vera van der Nulft, Arianne Stoppelenburg, Liesbeth M. van Vliet, Jenny T. van der Steen and Yvette M. van der Linden in Journal of Alzheimer's Disease

sj-pdf-2-alz-10.1177_13872877261461216 - Supplemental material for Communication strategies for improving readiness for advance care planning in dementia: A systematic review Supplemental material, sj-pdf-2-alz-10.1177_13872877261461216 for Communication strategies for improving readiness for advance care planning in dementia: A systematic review by Vera van der Nulft, Arianne Stoppelenburg, Liesbeth M. van Vliet, Jenny T. van der Steen and Yvette M. van der Linden in Journal of Alzheimer's Disease

sj-pdf-3-alz-10.1177_13872877261461216 - Supplemental material for Communication strategies for improving readiness for advance care planning in dementia: A systematic review Supplemental material, sj-pdf-3-alz-10.1177_13872877261461216 for Communication strategies for improving readiness for advance care planning in dementia: A systematic review by Vera van der Nulft, Arianne Stoppelenburg, Liesbeth M. van Vliet, Jenny T. van der Steen and Yvette M. van der Linden in Journal of Alzheimer's Disease

sj-pdf-4-alz-10.1177_13872877261461216 - Supplemental material for Communication strategies for improving readiness for advance care planning in dementia: A systematic review Supplemental material, sj-pdf-4-alz-10.1177_13872877261461216 for Communication strategies for improving readiness for advance care planning in dementia: A systematic review by Vera van der Nulft, Arianne Stoppelenburg, Liesbeth M. van Vliet, Jenny T. van der Steen and Yvette M. van der Linden in Journal of Alzheimer's Disease
