# Supplementary material for: Communication strategies for improving readiness for advance care planning in dementia: A systematic review
Source: J Alzheimers Dis. 2026 Jun 26;112(4):1735–50. doi: 10.1177/13872877261461216 (PMC13433894; doi:10.1177/13872877261461216)
Supplement: Supplementary file 1 [file sj-pdf-2-alz-10.1177_13872877261461216.pdf]

Supplemental Table 3. List of full-text screening of articles

Study information				Criteria (Yes/no)						Evaluation	
#	Authors	Year	Title	**Design:** Original empirical peer-reviewed study, including qualitative, quantitative, mixed methods, and observational studies, randomized control trials, and literature reviews	**Language:** English	**Sample:** People with dementia or cognitive impairment and/or family caregivers.	**Topic:** Includes communication strategies for ACP (any intervention incorporating communication-related components, such as scheduled conversations, training, education, and tools including manuals or conversation aids, or specific communication method used within ACP discussions, such as conversational techniques or interactional approaches).	**Outcome:** Includes 'readiness' or related concepts, consisting of willingness, receptiveness, acceptance, ability, attitude, preparedness or openness to engage in ACP	Inclusion (Yes/No)	Comments	Reason for exclusion
1	N. Ali; P. Anthony; W. S. Lim; M. S. Chong; E. W. H. Poon; V. Drury; M. Chan	2021	Exploring Differential Perceptions and Barriers to Advance Care Planning in Dementia among Asian Patient-Caregiver Dyads-A Mixed-Methods Study	Yes, parallel mixed methods	Yes	Yes	Yes, ACP counseling with a trained ACP facilitator	Yes	Yes		
2	R. S. Allen; S. R. DeLaine; W. F. Chaplin; D. C. Marson; M. S. Bourgeois; K. Dijkstra; L. D. Burgio	2003	Advance care planning in nursing homes: correlates of capacity and possession of advance directives	Yes, cross-sectional cohort	Yes	Yes, part of the sample		No	No		No readiness
3	L. L. Behrens; B. Morgan; E. Summerhayes; S. Talwar; V. L. Burshnic-Neal; M. Ersek; N. Hodgson	2022	Feasibility and acceptability of a web-based advance care plan for dementia	Yes, convergent mixed-methods	Yes	Yes	Yes, web-based decision aid	No	No		No readiness
4	B. S. Black; L. A. Fogarty; H. Phillips; T. Finucane; D. J. Loreck; A. Baker; D. M. Blass; P. V. Rabins	2009	Surrogate decision makers' understanding of dementia patients' prior wishes for end-of-life care	Yes, qualitative	Yes	Yes	No	No	No		No communication
5	S. R. Bolt; J. T. van der Steen; C. Khemai; J. M. G. A. Schols; S. M. G. Zwakhalen; J. M. M. Meijers	2022	The perspectives of people with dementia on their future, end of life and on being cared for by others: A qualitative study	Yes, qualitative	Yes	Yes	Yes (e.g. small talk,, adjusting oneés height, open and attentive posture)	No	No		No readiness
6	F. Bosisio; R. J. Jox; L. Jones; E. Rubli Truchard	2018	Planning ahead with dementia: what role can advance care planning play? A review on opportunities and challenges	Yes, review	Yes	Yes	No, communication only mentioned in general (no strategies)	No	No		No readiness
7	F. Bosisio; A.-C. Sterie; E. Rubli Truchard; R. J. Jox	2021	Implementing advance care planning in early dementia care: results and insights from a pilot interventional trial	Yes, pilot trial	Yes	Yes	Yes	No	No	Is not about readiness, more focused on advance directive documentation	No readiness
8	K. Brazil; G. Carter; C. Cardwell; M. Clarke; P. Hudson; K. Froggatt; D. McLaughlin; P. Passmore; W. G. Kernohan	2018	Effectiveness of advance care planning with family carers in dementia nursing homes: A paired cluster randomized controlled trial	Yes, RCT	Yes	Yes	Yes, ACP intervention	No	No		No readiness
9	G. Carter; D. McLaughlin; W. G. Kernohan; P. Hudson; M. Clarke; K. Froggatt; P. Passmore; K. Brazil	2018	The experiences and preparedness of family carers for best interest decision-making of a relative living with advanced dementia: A qualitative study	Yes, qualitative	Yes	Yes	No, not about ACP	Yes, preparedness	No		No ACP
10	A. G. Catic; A. I. Berg; J. A. Moran; J. R. Knopp; J. L. Givens; D. K. Kiely; N. Quinlan; S. L. Mitchell	2013	Preliminary data from an advanced dementia consult service: Integrating research, education, and clinical expertise	Yes, quantitative	Yes	No	Yes, "Advanced Dementia Consult Service" (with ACP components)	No	No		No readiness
11	W. C. H. Chan; T. K. Yu	2021	Conducting an advance care planning group among older adults living in residential care homes: An initiative of social workers in Hong Kong	Yes	Yes	No	No, communication as an outcome but no mention of strategies	No, focus on willingness, but that of advance directives	No	Sample is frail elderly (no dementia or cognitive impairment)	Sample
12	D. R. Chen; H. C. Weng	2023	Associations of health literacy, personality traits, and pro-individualism with the willingness to complete advance directives in Taiwan	Yes, quantitative	Yes	Yes	No	No	No	Is about on factors for readiness, but does not cover any communication strategies	No communication
13	L. Chen; G. Yin; S. Lin; Y. Li	2022	The perspectives of family caregivers of people with Alzheimer's disease regarding advance care planning in China: A qualitative research	Yes, qualitative	Yes	Yes	No	Yes, somewhat	No	Is partially focused on factors for readiness, but does not cover any communication strategies	No communication
14	M. A. Clark; M. Ott; M. L. Rogers; M. C. Politi; S. C. Miller; L. Moynihan; K. Robison; A. Stuckey; D. Dizon	2017	Advance care planning as a shared endeavor: completion of ACP documents in a multidisciplinary cancer program	Yes, observational	Yes	No	No, focus on documentation	No	No		Sample
15	G. Clarke; E. Fistein; A. Holland; J. Tobin; S. Barclay; S. Barclay	2018	Planning for an uncertain future in progressive neurological disease: a qualitative study of patient and family decision-making with a focus on eating and drinking	Yes, qualiative	Yes	Yes (n=4 of 34 participants)	No	Yes	No	It is about what you need for making decisions, not focused on readiness (and parts that mention readiness is too general/not an outcome)	No communication
16	C. Clavien; C. Schopfer; C. Bollondi; J. Sommer	2023	Effect of an App for Promoting Advance Care Planning and Motivating Patients to Write Their Advance Directives	Yes, quantative	Yes	No, unclear what kind of patients (ambulatory patients in general)	Yes, an app	Yes	No	Unclear if people with dementia are included	Sample
17	T. K. M. Cudjoe; C. M. Boyd; J. L. Wolff; D. L. Roth	2020	Advance Care Planning: Social Isolation Matters	Yes, quantitative (survey)	Yes	Yes	No	No	No	Is not about readiness for ACP, but about markers for ACP and if that can be predicted based on social isolation	No readiness
18	N. Davies; N. Aker; V. Vickerstaff; E. L. Sampson; G. Rait	2022	A feasibility study of a decision aid to support family carers of people with severe dementia or those towards the end-of-life	Yes, pretest-posttest design	Yes	Yes, family caregivers of people with severe dementia	Yes, decision aid	No	No	Is about use of advance directive to have less decisional conflict and distress, and more satisfactin of care en quality of life, but not readiness	No readiness
19	N. Davies; T. De Souza; G. Rait; J. Meehan; E. L. Sampson	2021	Developing an applied model for making decisions towards the end of life about care for someone with dementia	Yes	Yes	Yes	No, it is about the process of decision-making (not ACP necessarily)	No	No	It is about process of decision-making instead of ACP specifically	No ACP
20	N. Davies; J. Manthorpe; E. L. Sampson; K. Lamahewa; J. Wilcock; R. Mathew; S. Iliffe	2018	Guiding practitioners through end of life care for people with dementia: The use of heuristics	Yes	Yes	No, sample is healthcare professionals	No, not about ACP	No	No	Qualitative evalation of the use of heuristics to discuss important topics (e.g. swallowing). This data is about clinicians. And also no focus on readiness.	Sample
21	M. E. de Boer; R.-M. Dröes; C. Jonker; J. A. Eefsting; C. M. P. M. Hertogh	2012	Thoughts on the Future: The Perspectives of Elderly People with Early-Stage Alzheimer's Disease and the Implications for Advance Care Planning	Yes	Yes	Yes	No	No	No	Qualitative study with people with early Alzheimer's disease. It is about how they feel about the future and ACP, not about communication and readiness.	No communication
22	K. de Vries; J. Drury-Ruddlesden	2019	Advance care planning for people with dementia: Ordinary everyday conversations	Yes	Yes	Yes, family caregivers of deceased PwD	Yes (e.g. ordinary everyday conversations, knowing the person, communication about unexpected encounters)	No	No	Qualiative study of family caregivers' experiences of how decisions for their deceased loved ones were made	No readiness
23	K. S. Deep; A. Hunter; K. Murphy; A. Volandes	2010	It helps me see with my heart': How video informs patients' rationale for decisions about future care in advanced dementia	Yes	Yes	No	Yes, somewhat through video	No	No	It is an experimental video-vignette study on the effect of a video on care preferences, but using a hypothethical sample (not people with dementia or family caregivers)	Sample
24	J. deLima Thomas; S. Sanchez-Reilly; R. Bernacki; L. O'Neill; L. J. Morrison; J. Kapo; V. S. Periyakoil; E. C. Carey	2018	Advance Care Planning in Cognitively Impaired Older Adults	No, overview article (not a review)	Yes	No	Yes, some interventions mentioned	No	No	Overview article. It does contain interesting information on interventions and tools to aid ACP	Design
25	D. Dempsey	2013	Advance care planning for people with dementia: Benefits and challenges	No, overview article (not a review)	Yes	No	No	No	No	It is an overview article of ACP. It does state the importance of communication herein, but it's not an empirical study into readiness.	Design
26	C. R. Durbin; A. F. Fish; J. A. Bachman; K. V. Smith	2010	Systematic review of educational interventions for improving advance directive completion	Yes, systematic review	Yes	yes	No	No, it's about completion of advance directives, not readiness	No		No readiness
27	M. R. Ekkel; M. Depla; E. M. L. Verschuur; R. B. Veenhuizen; C. Hertogh; B. D. Onwuteaka-Philipsen	2022	Patient perspectives on advance euthanasia directives in Huntington's disease. A qualitative interview study	Yes	Yes	No, huntington disease patients	No	No	No	Sample is Huntington disease patients' views on advance euthanasie directives (not dementia or focused on cognitive impairment)	Sample
28	M. Eneslätt; G. Helgesson; C. Tishelman	2021	Dissemination, use, and impact of a community-based, conversational advance care planning intervention: ripple effects of the Swedish DöBra cards	Yes	Yes	No	Yes, use of tools (cards) to discuss end-of-life care	No	No	It is a study about how certain cards to discuss EOL preferences/priorities for general public are used in the community. Unclear to what extent the cases that were used (patient organization) are focused on dementia	Sample
29	C. N. Flaherty; P. A. Carter; L. Smith; N. Lerner; G. Hooper; J. R. Bail	2023	Community-based early dementia advance care planning in the United States: A scoping review	Yes, scoping review	Yes	Yes	No	No, no specific focus on readiness	Check included articles (see below)	Included articles of this scoping review were checked for inclusion. All are about facilitators for ACP, but too general and not focused on readiness as an outcome	No readiness
30	B. Francesca; J. Jox Ralf; J. Laura; T. E. Rubli	2018	Planning ahead with dementia: What role can advance care planning play? A review of opportunities and challenges	No, overview article (not a review)	Yes	Yes	Yes	No	No	Does adress challenges of ACP and how to overcome them (e.g. know whom to talk to, have a window of opportunity etc), but not an empirical study	Design
31	T. R. Fried; A. B. Cohen; J. E. Harris; L. Moreines	2021	Cognitively Impaired Older Persons' and Caregivers' Perspectives on Dementia-Specific Advance Care Planning	Yes, qualitative	Yes	Yes	No, the results focus on barriers and not how to overcome them	Yes	No	This qualitative study focuses on barriers for ACP, but lacks how to overcome these barriers and a focus on readiness.	No communication
32	A. Geddis-Regan; L. Errington; C. Abley; R. Wassall; C. Exley; R. Thomson	2021	Enhancing shared and surrogate decision making for people living with dementia: A systematic review of the effectiveness of interventions	Yes, systematic review	Yes	Yes	Yes	No	No	Is about improvement of shared decision making in dementia in general. Partially about ACP, but the included interventions are mainly measured with satisfaction with communication, quality of communication and less decision-making conflict after (no readiness)	No readiness
33	J. L. Givens; R. L. Sudore; G. A. Marshall; A. B. Dufour; I. Kopits; S. L. Mitchell	2018	Advance Care Planning in Community-Dwelling Patients With Dementia	Yes, survey	Yes	Yes	No	Yes	No	Survey study about views of proxies of dementia patients, including if they would like to have information about end-stage dementia (defined as readiness in the article), but no specific mention of communication strategies	No communication
34	B. Goossens; A. Sevenants; A. Declercq; C. Van Audenhove	2020	Improving shared decision-making in advance care planning: Implementation of a cluster randomized staff intervention in dementia care	Yes	Yes	No	Yes	No	No	Study focused on healthcare professionals; how an intervention can increase shared decisiuon-making in ACP, the perceived importance of shared decision-making, and facilitating/hindering elements for sustanability of training.	Sample
35	C. F. Hamel; L. W. Guse; P. G. Hawranik; J. B. Bond, Jr.	2002	Advance directives and community-dwelling older adults	Yes, experimental	Yes	No	Yes, discussion of advance directives and completion of them	No	No		Sample
36	K. B. Hirschman; J. M. Kapo; J. H. T. Karlawish	2008	Identifying the factors that facilitate or hinder advance planning by persons with dementia	Yes, qualitative	Yes	Yes, family caregivers	No, is about facilitators in general (no focus on communication strategies)	No	No	Readiness is mentioned once in general (no outcome), and no communication strategies are mentioned (prompts for ACP included discussion with attorney and healthcare professional, but it was not mentioned what kind of discussion).	No readiness
37	H.-L. Huang; W.-R. Lu; C.-L. Liu; H.-J. Chang	2020	Advance care planning information intervention for persons with mild dementia and their family caregivers: Impact on end-of life care decision conflicts	Yes, quantitative	Yes	Yes	Yes, an ACP information intervention	Yes, positive or negative attitude towards ACP	Yes	Evaluation of ACP intervention (delivered by nurse, including information about e.g. medical treatments and benefits/risks family-centered strategy for ACP and faciliatorn of open discussion); with influence on attitude as an outcome	
38	I. Kermel Schiffman; P. Werner	2021	Willingness of family caregivers of people with dementia to undertake Advance Care Planning: Examining an extended model of the Theory of Planned Behavior	Yes	Yes	Yes	No	Yes	No	Study about predictors of willingness for ACP, but these predictors are not about communication (e.g. about attitude towards ACP, subjective norms, prolonging life, etc.)	No communication
39	E. Ko; M. Hohman; J. Lee; A. N. Ngo; S. I. Woodruff	2016	Feasibility and Acceptability of a Brief Motivational Stage-Tailored Intervention to Advance Care Planning: A Pilot Study	Yes, qualitative	Yes	No	Yes, motivational interviewing intervention	Yes	No	No the right sample; although it is about an motivational interviewing intervention that might improve stage of change (readiness) towards ACP	Sample
40	E. Ko; J. Lee; C. Ramirez; D. Lopez; S. Martinez	2018	Patient-family EoL communication and its predictors: Reports from caregivers of Latino patients in the rural U.S.-Mexico border region	Yes, survey	Yes	No	No, more generally focused on factors associated with communication (not strategies)	No	No		Sample
41	M. Lai; Y.-H. Jeon; H. McKenzie	2019	The key factors for the engagement of primary stakeholders in decision-making for the future care of people with dementia living in the community: A systematic integrative review	Yes, review	Yes	Yes	No	Maybe, factors for engagement for decision-making, but nothing specific about communication; check for included articles	Check included articles (see below)	Included articles of this review were checked for inclusion. However, included articles are about readiness in general, not about communication	No communication
42	L. Lee; L. M. Hillier; S. K. Lu; D. Ward	2021	Enabling Advance Care Planning in Dementia Care: A Primary Care Approach	Yes, mixed mehtods studie	Yes	Yes	Yes, ACP framework tool	No	No	The outcome is facilitation of ACP discussions and not readiness	No readiness

43	N. Lemos Dekker; S. R. Bolt	2022	Relating to the end of life through advance care planning: Expectations and experiences of people with dementia and their family caregivers	Yes, qualitative	Yes	Yes	No	Yes, some mention of readiness and barriers related to avoidance	No	Is partly about readiness, but not related to communication	No communication
44	H. D. Lum; D. E. Barnes; M. T. Katen; Y. Shi; J. Boscardin; R. L. Sudore	2018	Improving a Full Range of Advance Care Planning Behavior Change and Action Domains: The PREPARE Randomized Trial	Yes, quantitative	Yes	No	Yes, a tool (website)	Yes	No		Sample
45	H. D. Lum; S. R. Jordan; A. Brungardt; R. Ayele; M. Katz; J. M. Miyasaki; A. Hall; J. Jones; B. Kluger	2019	Framing advance care planning in Parkinson disease: Patient and care partner perspectives	Yes, qualitative	Yes	No	No	No, readiness only mentioned in discussion	No	Is about Parkinsons disease, but not Parkinsons dementia or cognitive impairment	Sample
46	Z. A. Macchi; H. D. Lum	2022	Advance care planning in neurologic illness	No, handbook	Yes	No, dementia mentioned but not as participants	Yes, interventions for Acp in dementia are mentioned	No, readiness only mentioned in abstract	No		Design
47	R. D. McMahan; I. Tellez; R. L. Sudore	2021	Deconstructing the Complexities of Advance Care Planning Outcomes: What Do We Know and Where Do We Go? A Scoping Review	Yes, review	Yes	Yes	Yes, communication mentioned a lot	Yes, includes references to articles focused on readiness/preparedness, but sample not clear; check for included articles	Check included articles (see below)	Included articles of this review were checked for inclusion. All included articles that focused on readiness included different samples (no dementia)	Sample
48	K. J. Moore; H. Goodison; E. L. Sampson	2019	The role of the memory service in helping carers to prepare for end of life: A mixed methods study	Yes, mixed methods	Yes	No, clinicians	Yes	No, not about readiness for ACP	No	Wrong sample, and is also not about how the services influence readiness for ACP	Sample
49	L. Murray; P. N. Butow; K. White; M. C. Kiernan; N. D'Abrew; H. Herz	2016	Advance care planning in motor neuron disease: A qualitative study of caregiver perspectives	Yes, qualitative	Yes	No, motor neuron disease	Yes, results mention some communication stratgies (e.g. initiation)	No, focused on readiness for death (not ACP)	No	Not focused on sample with cognitive impairment/dementia. Also not focused on readiness for ACP, but readiness for voor death	Sample
50	S. J. Oczkowski; H. O. Chung; L. Hanvey; L. Mbuagbaw; J. J. You	2016	Communication tools for end-of-life decision-making in the intensive care unit: a systematic review and meta-analysis	Yes, review	Yes	No	Yes, communication tools	No	No	No dementia sample	Sample
51	S. Orsulic-Jeras; C. J. Whitlatch; S. M. Szabo; E. G. Shelton; J. Johnson	2019	The SHARE program for dementia: Implementation of an early-stage dyadic care-planning intervention	Yes qualitative	Yes	Yes	Yes, intervention (SHARE)	No	No	No outcome of readiness	No readiness
52	K. Ouchi; C. Joshi; J. Kaithamattam; S. A. Gale; G. A. Marshall; A. Pietras; W. Wang; E. W. Boyer; J. A. Tulsky; S. D. Block; D. Rentz; M. A. Schonberg	2024	Refinement of an Emergency Department-Based, Advance Care Planning Intervention for Patients With Cognitive Impairment and Their Caregivers	Yes, quantativie	Yes	Yes	Yes, ED Goal	Yes	Yes		
53	F. B. A. L. Peerboom; J. H. H. M. Friesen-Storms; B. J. E. G. Coenegracht; S. Pieters; J. T. van der Steen; D. J. A. Janssen; J. M. M. Meijers	2023	Fundamentals of end-of-life communication as part of advance care planning from the perspective of nursing staff, older people, and family caregivers: a scoping review	Yes, review	Yes	Yes, both PwD and FCGs	No	Yes	No	Readiness is mentioned a lot, but more generally (e.g. importance of readiness); not focused on communication strategies	No communication
54	M. Poppe; S. Burleigh; S. Banerjee	2013	Qualitative evaluation of advanced care planning in early dementia (ACP-ED)	Yes, qualitative	Yes	Yes	Yes	No	No	Includes readiness in the text, but not as an outcome	No readiness
55	T. J. L. Sævareid; L. Thoresen; E. Gjerberg; L. Lillemoen; R. Pedersen	2019	Improved patient participation through advance care planning in nursing homes—A cluster randomized clinical trial	Yes, cluster RCT	Yes	Yes, part of the sample	No, only mentioned generally as a barrier (lackk of skills)	No	No	Readiness is not mentioned, no outcome	No communication
56	J. B. Sinclair; J. R. Oyebode; R. G. Owens	2016	Consensus views on advance care planning for dementia: a Delphi study	Yes, Delphi study	Yes	Yes	No, only some tools mentioned in recommendation (not the main focus)	No, only mentioned in abstract	No		No communication
57	M. K. Song; S. E. Ward; K. Hepburn; S. Paul; H. Kim; R. C. Shah; D. J. Morhardt; L. Medders; J. J. Lah; C. C. Clevenger	2019	Can Persons with Dementia Meaningfully Participate in Advance Care Planning Discussions? A Mixed-Methods Study of SPIRIT	Yes, mixed methods	Yes	Yes	Yes, Spirit intervention	No	No	Readiness is only mentioned generally in the discussion (no outcome)	No readiness
58	F. Stewart; C. Goddard; R. Schiff; S. Hall	2011	Advanced care planning in care homes for older people: a qualitative study of the views of care staff and families	Yes, qualitative	Yes	No, sample is staff (care managers, nurses and care assistants), community nurses and families of care home residents (not dementia-specific)	No	No	No	Is about barriers and facilitators for ACP in general, not about readiness	Sample
59	T. Sussman; J. Lawrence; R. Pimienta	2022	This is how I want it": Exploring the use of a workbook with persons with dementia to support advance care planning engagement"	Yes, mixed method	Yes	Yes, both PwD and FCGs	Yes, a workbook	Yes	Yes		
60	T. Sussman; R. Pimienta; A. Hayward	2021	Engaging persons with dementia in advance care planning: Challenges and opportunities	Yes, qualitative	Yes	Yes, both PwD and FCGs	No, only some tools mentioned in recommendation (not the main focus)	No	No		No readiness
61	S.Y. Tay; J. Davison; N.C. Jin; P.L.K. Yap	2015	Education and Executive Function Mediate Engagement in Advance Care Planning in Early Cognitive Impairment	Yes, quantative	Yes	Yes	No	Yes	No	Not about communication	No communication
62	R. Van Rickstal; A. De Vleminck; M. D. Aldridge; S. R. Morrison; R. T. Koopmans; J. T. van der Steen; S. Engelborghs; L. Van den Block	2019	Limited engagement in, yet clear preferences for advance care planning in young-onset dementia: An exploratory interview-study with family caregivers	Yes, qualitative	Yes	Relativces of patients with young-onset dementie	Yes	Yes	Yes		
63	R. Van Rickstal; A. De Vleminck; S. Engelborghs; J. Versijpt; L. Van den Block	2022	A qualitative study with people with young-onset dementia and their family caregivers on advance care planning: A holistic, flexible, and relational approach is recommended	Yes, qualitative	Yes	Yes, young-onset dementia patients and family caregivers	No	No	No	Only generally refers to readiness in discussion (not an outcome)	No communication
64	R. Van Rickstal; A. De Vleminck; S. R. Morrison; R. T. Koopmans; J. T. van der Steen; S. Engelborghs; J. Neugroschl; M. D. Aldridge; M. Sano; L. Van den Block	2020	Comparing Advance Care Planning in Young-Onset Dementia in the USA vs Belgium: Challenges Partly Related to Societal Context	Yes qualitative	Yes	Yes	No, more focused on barriers and limited communication in general (no strategies)	No	No	Only generally refers to readiness in discussion (not an outcome)	No communication
65	M. C. van Soest-Poortvliet; J. T. van der Steen; G. Gutschow; L. Deliens; B. D. Onwuteaka-Philipsen; H. C. de Vet; C. M. Hertogh	2015	Advance Care Planning in Nursing Home Patients With Dementia: A Qualitative Interview Study Among Family and Professional Caregivers	Yes, qualitative	Yes	Yes, PwD, FCGs, and healthcare professionals	No	No	No	Not about readiness (only very generally in the discussion) and no communication strategies	No readiness

66	V.T. Cotter; M.M. Hasan; J. Ahn; C. Budhathoki; E. Oh	2019	A Practice Improvement Project to Increase Advance Care Planning in a Dementia Specialty Practice	Yes	Yes	No, physicians	No, more focused on self-efficacy to discuss ACP (no communication strategies)	No	No	Focus on healthcare professionals	Sample
67	J. Gabbard; D. Johnson; G. Russel	2020	Prognostic Awareness, Disease and Palliative Understanding Among Caregivers of Patients With Dementia	Yes	Yes	Yes	No, it is about prognostic understanding	Yes, does include some questions about readiness for palliative care (e.g. do you want education for future problems)	No	It is about prognostic understanding and readiness for palliative care	No ACP
68	K.L. Harrison; J.H. Bull; S.B. Garrett; L. Bonsingnore; T. Bice; L.C. Hanson; C.S. Richie	2020	Community-Based Palliative Care Consultations: Comparing Dementia to Nondementia Serious Illnesses	Yes	Yes	Yes	No, focused on prognosis and advance directives (not discussing ACP)	No	No	It is a study describing the difference between dementia and non-dementia patients who receive a palliative care consultation	No ACP
69	T. Fried; K. Bullock; L. Iannone; J.R. O'Leary	2009	Understanding Advance Care Planning as a Process of Health Behavior Change	Yes	Yes	No, community-dwelling older people and family caregivers. No mention of dementia	No, communication only broadly mentioned (no strategies)	Yes, health behavior change	No	No focus on dementia - it is a qualitative study about which strategies they use to increase readiness, e.g. individualizing communication to the need of a loved one.	Sample
70	J. Kwak; B. Xie; J.D. Champion; K.R. Fleischmann	2019	Rural Dementia Caregivers in Southwest Texas: An Exploratory Study of Advance Directives and End-of-Life Proxy Decision Making	Yes	Yes	Yes	No, not focused on ACP	No	No		No ACP
71	A. Shafir; C.S. Richie; S.B. Garrett; S.A. Bernstein; G. Naasan; J. Merrilees; E. Widera; L. Flint; K.L. Harrison	2020	"Captive by the Uncertainty" - Experiences with Anticipatory Guidance for People Living with Dementia and Their Caregivers at a Specialty Dementia Clinic	Yes	Yes	Yes	No, it is about what patients and family caregivers need from clinicians (e.g. more information about what to come)	No	No	Qualitative study on what patients and caregivers need from dementia clinics in general	No readiness
72	K. Dassel; R. Utz; K. Supiano; S. Bybee; E. Iacob	2019	Development of a Dementia-Focused End-of-Life Planning Tool: The LEAD Guide (Life-Planning in Early Alzheimer's and Dementia	Yes	Yes	Yes	Yes, development and first evaluation of a tool about EoL preferences	No	No	It is about the development of a tool for patients with ADRD that provides information about their care preferences, which family caregives can use later on to make decisions; not about readiness	No readiness
73	M.J. Karel; J. Moye, A. Bank; A.R. Azar	2007	Three Methods of Assessing Values for Advance Care Planning: Comparing Persons With and Without Dementia	Yes	Yes	Yes	Yes, healthcare values assessment tools	No, it is about utility for healthcare values assessment tool for people with dementia.	No	It is about utility of a health assessment tool; no focus on readiness	No readiness
74	G.J. Bonner; S. Freels; D.J. Wilie; et al.	2020	Advance Care Planning for African American Caregivers of Relatives With Dementias: Cluster Randomized Controlled Trial	Yes	Yes	Yes, African American family caregivers	Yes, intervention to increase knowledge and self-efficacy about EOL treatment decisions	No	No	Is about intention to make a care plan	No readiness
75	M.M. Hilgeman; R.S. Allen; L. Snow; D.W. Dukin; J. DeCoster; L. Burgio	2013	Preserving Identity and Planning for Advance Care (PIPAC): preliminary outcomes from a patient-centered intervention for individuals with mild dementia	Yes	Yes	Yes, mild dementia and family caregivers	Yes, intervention	No	No	Decision conflict as an outcome (not readiness)	No readiness
76	L. Garand; M.A. Dew; J.H. Lingler; S.T. DeKosky	2011	Incidence and Predictors of Advance Care Planning Among Persons With Cognitive Impairment	Yes	Yes	Yes	No, study about predictors of ACP status (clinical and demographic data)	No	No		No readiness
77	J.H. Lingler; K.B. Hierschman; L. Garand; J.T. Becker; R. Schulz; S.T. Dekosy	2008	Frequency and Correlates of Advance Planning Among Cognitively Impaired Older Adults	Yes	Yes	Yes	No, study is about predictors of written advance care planning (durable power of attorney and living will status)	No	No		No readiness
78	K. Beernaert; L. Deliens; A. De Vleminck; D. Devroey; K. Pardon; L. Van den Block1; J. Cohen	2014	Early identification of palliative care needs by family physicians: a qualitative study of barriers and facilitators from the perspective of family physicians, community nurses, and patients	Yes	Yes	Yes, but also other diseases	No, it is about palliative care needs, not ACP	No	No	It is about identification of EoL needs, not about ACP	No ACP
79	K. Bronner 1; R. Perneczky; R. McCabe; A. Kurz ; J. Hamann	2016	Which medical and social decision topics are important after early diagnosis of Alzheimer's disease from the perspectives of people with Alzheimer's disease, spouses and professionals?	Yes	Yes	Yes	Not about ACP (focused on davance directives)	No	No	Not explicitly about readiness	No readiness
80	M.E. de Boer; R. Dröes; C. Jonker; J.A. Eefsting; C.M.P.M. Hertogh	2012	Thoughts on the future: the perspectives of elderly people with early-stage Alzheimer's disease and the implications for advance care planning	Yes	Yes	Yes	No	No	No	Not focused on communication strategies	No communication
81	A. De Vleminck; K. Pardon; K. Beernaert; D. Houttekier; R Van der Stichele; L. Deliens	2014	How do general practitioners conceptualise advance care planning in their practice?	Yes	Yes	No	No, some mention of communication (but very general)	No, willingness shortly mentioned (but in general)	No	Not about dementia, and also not about readiness	Sample
82	K.H. Dening; L. Jones; E.L. Sampson	2013	Preferences for end-of-life care: a nominal group study of people with dementia and their family carers	Yes	Yes	Yes	No	No	No	Not about readiness	No communication
83	K.H. Dening; M. King; L. Jones; E.L. Sampson	2017	Healthcare decision-making: past present and future, in light of a diagnosis of dementia	Yes	Yes	Yes	No	No	No	Concerns decision making but not readiness	No communication
84	C. Dickinson; C. Bamford; C. Exley; C. Emmett; J. Hughes; L. Robinson	2013	Planning for tomorrow whilst living for today: the views of people with dementia and their families on advance care planning.	Yes	Yes	Yes	No	No, only generally mentioned once	No	Not focused on communication strategies; does give implications in the conclusion (timing and informal discussions), but not main focus of the study (too general)	No communication
85	C. Drummond; A. Simpson	2017	Who's actually gonna read this?' An evaluation of staff experiences of the value of information contained in written care plans in supporting care in three different dementia care settings	Yes	Yes	No	No	No	No	Aim is to investigate the experiences and views of staff in relation to care planning in dementia	Sample
86	D. Fetherstonhaugh; L. Tarzia; R. Nay	2013	Being central to decision making means I am still here!: the essence of decision making for people with dementia	Yes	Yes	Yes	No	No	No	It is not about ACP or readiness	No ACP
87	A. O'kelly; G. Howarth; G. Richards; T. Butcher	2015	Advance planning for end of life care	Yes	Yes	Yes	No, not about ACP	No	No	It is not about ACP or readiness	No ACP
88	M. Poole; C. Bamford; E. McLellan; R.P. Lee; C. Exley; J.C. Hughes; K.H. Dening; L. Robinson	2018	End-of-life care: a qualitative study comparing the views of people with dementia and family carers	Yes	Yes	Yes	No, some mention of communication (but very general)	No	No	It is about discussing optimal EoL care in general, not readiness	No communication
89	M. Poppe; S. Burleigh	2013	Qualitative evaluation of advanced care planning in early dementia (ACP-ED).	Yes	Yes	Yes	No, is about challenges in general (no communication)	Yes	No	Is more about challenges with ACP in general; Readiness is mentioned, but not in relation to communication strategies	No communication
90	L. Robinson; C. Dickinson; C. Bamford; A. Clark; J. Hughes; C. Exley	2013	A qualitative study: Professionals' experiences of advance care planning in dementia and palliative care, 'a good idea in theory but ...'	Yes	Yes	No, HCPs	No	No	No	Focused on healthcare professionals	Sample
91	T. Ryan; C. Gardiner; G. Bellamy; M. Gott; C. Ingleton	2012	Barriers and facilitators to the receipt of palliative care for people with dementia: the views of medical and nursing staff	Yes	Yes	No, HCPs	No	No	No	Focused on healthcare professionals	Sample
92	K. Brazil; G. Carter; K. Galway; M. Watson; J.T. van der Steen	2015	General practitioners perceptions on advance care planning for patients living with dementia.	Yes	Yes	No, HCPs	No	Yes, willingness	No	Focused on healthcare professionals	Sample
93	G. Carter; J.T. van der Steen; K. Galway; K. Brazil	2017	General practitioners' perceptions of the barriers and solutions to good-quality palliative care in dementia	Yes	Yes	No, GPs	No	No	No		Sample
94	M. Lewis; E. Rand; E. Mullaly; D. Mellor; S. Macfarlane	2015	Uptake of a newly implemented advance care planning program in a dementia diagnostic service.	Yes	Yes	Yes	Yes	No	No		No readiness
95	K. Cheong; P. Fisher; J. Goh; L. Ng; H.M. Koh; P. Yap	2015	Advance care planning in people with early cognitive impairment	Yes	Yes	Yes	No	Yes	No	Is about readiness (willingness), but not about communication strategies that infleunce it; focused on barriers	No communication
96	S.R. Hill; H. Mason; M. Poole; L. Vale; L. Robinson	2017	What is important at the end of life for people with dementia? The views of people with dementia and their carers	Yes	Yes	Yes	No, not about ACP	No	No		No ACP
97	Y.H. Yun; E. Kang; S. Park S; et al.	2019	Efficacy of a decision aid consisting of avideo and booklet on advance care planning for advanced cancerpatients: randomized controlled trial	Yes	Yes	No	Yes, decision aid	No, focus on documentation of ACP	No		Sample
98	T.J.L. Sævareid; L. Thoresen; E. Gjerberg; L. Lillemoen; R. Pedersen	2019	Improved patient participation through advance care planning in nursing homes: a cluster randomized clinical trial	Yes	Yes	No	Yes	No, focus on documentation of ACP	No		Sample
99	M.K. Song; S.E. Ward; F.C. Lin; et al.	2017	Racial differences in outcomes of an advance care planning intervention for dialysis patients and their surrogates	Yes	Yes	No	Yes	No	No		Sample
100	V.J. Periyakoil	2018	A randomized clinical feasibility trial comparing the letterproject advance directive to traditional advance directive	Yes	Yes	No	No, focused on understanding advance directives	No	No		Sample
101	H.D. Lum; D.E. Barnes; M.T. Katen; Y. Shi; J. Boscardin; R.L. Sudore	2015	Improving a full range of advance care planning behavior change and action domains: the PREPARE randomized trial	Yes	Yes	No	Yes	Yes	No		Sample
102	R.L. Sudore; D. Schillinger; M.T. Katen; et al.	2018	Engaging diverse English- and Spanish-speaking older adults in advance care planning: the PREPARE randomized clinical trial	Yes	Yes	No	Yes, PREPARE intervention	Yes	No		Sample

103	R. Sudore; G.M. Le; R. McMahan; M. Feuz; M. Katen; D.E. Barnes	2015	Community-Based Palliative Care Consultations: Comparing Dementia to Nondementia Serious Illnesses		Yes	Yes	No	No	No		No		Sample
104	C.H. Chiu Wu; S.J. Perng; C.K. Shi; H.L. Lai	2019	Advance care planning and advance directives: a multimedia education program in community-dwellingolder adults		Yes	Yes	No	Yes, education intervention	Yes, willingness mentioned		No		Sample
105	B. Goossens; A. Sevenants; A. Declercq; C. Van Audenhove	2019	Improving shared decision-making in advance care planning: implementation of a cluster ran-domized staff intervention in dementia care		Yes	Yes	No	Yes, intervention	No, focused on shared decision-making		No		No readiness
106	J. Gilissen; L. Pivodic; A. Wendrich-van Dael; W. Cools; R. Van der Stichele; L. Van den Block; L. Deliens; C. Gastmans	2020	Nurses' self-efficacy, rather than their knowledge, is associated with their engagement in advance care planning in nursing homes: A survey study		Yes	Yes	No, nurses	No	No		No	Focused on nurses	Sample
107	C.C. Yeung; K.H. Ho; H.Y. Chan	2023	A dyadic advance care planning intervention for people with early-stage dementia and their family caregivers in a community care setting: a feasibility trial		Yes	Yes	Yes	Yes	Yes		Yes		
108	J.L.Wolff; J.G. Cagle; V. Hanna; S.M. Dy; D. Echavarria; E.R. Giovannetti; C.M. Boyd; M. Abshire Saylor; N. Hussain; J.S. Reiff; D. Scerpella; T. Zhang; V. Kaur Sekhon; D.L. Roth	2024	Sharing health carewishes among older adultswith cognitive impairment in primary care: Results from a randomized controlled trial		Yes	Yes	Yes	Yes	Yes		Yes		
109	M. Abshire Saylor; V. Hanna; P. Zhang; G. Huynh Thai; C. Mulholland Green; J.G. Cagle; J.L.Wolff	2024	Advance care planning in adults ages 80 years and older with impaired cognition: Using actual conversations to examine best practices		Yes	Yes	Yes	Yes	Yes		Yes		
110	C. Dupont; T. Smets; F. Monnet; L. Pivodic; A. De Vleminck; C. Van Audenhove; L. Van den Block	2024	A website to support people with dementia and their family caregivers in advance care planning: Results of a mixed-method evaluation study		Yes	Yes	Yes	Yes	Yes		Yes		
111	S. Vellani; M. Puts; A. Iaboni; K.S. McGilton	2022	Voice your values, a tailored advance care planning intervention in persons living with mild dementia: A pilot study		Yes	Yes	Yes	Yes	Yes		Yes		
