# Supplementary material for: Communication strategies for improving readiness for advance care planning in dementia: A systematic review
Source: J Alzheimers Dis. 2026 Jun 26;112(4):1735–50. doi: 10.1177/13872877261461216 (PMC13433894; doi:10.1177/13872877261461216)
Supplement: Supplementary file 2 [file sj-pdf-3-alz-10.1177_13872877261461216.pdf]

Supplemental Table 4. Quantitative data extraction sheets of included studies

								a=Patients<br>b=Family<br>c=Healthcare staff<br>d=Mix (specify)				GP=General practice<br>HC=Home care<br>HO=Hospital<br>NH=Nursing home<br>OTH=other								a=Patient level<br>b= Caregiver level<br>c=Healthcare staff<br>d=Mix						a=Increased readiness<br>b=Decreased readiness<br>c=No difference<br>d=No (no intervention)		
Study information							Participants						Study methods						Intervention				Outcomes				Conclusions	Reviewer comments
#	Author(s)	Title	Journal	Year	Research aim	Research questions	Participants	Participants	N	Country	Setting	Setting	Design	Methods of data collection	Recruitment/sampling procedures	Randomization methods	Missing data handling	Statistical analyses	Intervention description	Intervention target	Readiness definition	Control group	Outcome Measures	Measurement Instrument	Results	Outcome classification	Author conclusions	
1	N. Ali; P. Anthony; W. S. Lim; M. S. Chong; E. W. H. Poon; V. Drury; M. Chan	Exploring Differential Perceptions and Barriers to Advance Care Planning in Dementia among Asian Patient-Caregiver Dyads-A Mixed-Methods Study	Int J Environ Res Public Health	2021	(a) Examine the disparity in perceptions among subjects with mild dementia and their caregivers towards ACP after undergoing ACP counseling and (b) Explore the barriers to ACP in dementia.	(Not mentioned)	Patient-caregiver dyads; Patients with mild dementia and their caregivers	d (a & b)	N=40 (n=20 dyads)	Singapore	Memory clinic	HO	Parallel mixed-method	Pre-/post-intervention questionnaires and post-intervention interviews)	Recruitment from outpatient attendees of the Memory Clinic at a tertiary hospital over a period of 2.5 years.<br><br>Inclusion criteria: (1) Newly diagnosed with dementia based on the Diagnostic Statistical Manual IV criteria; (2) Mild severity, as defined by a global Clinical Dementia Rating score (CDR) of 0.5–1; (3) No significant mood disorders or behavioral problems; (4) Had an identified caregiver who was conversant in English.<br><br>Exclusion criteria: (1) Moderate or severe dementia (CDR ≥ 2); (2) Who had a concomitant diagnosis of delirium; (3) Those with unstable medical conditions, defined as more than 2 hospitalizations in the last 1 month prior to recruitment.	No randomization	(Not mentioned)	(1) Descriptive analyses of the baseline patient and caregiver characteristics and (2) Univariate analysis by Fisher's exact test to determine the differences between the patient-caregiver dyads' responses to the questionnaires (the end-of-life communication between patients and caregivers).<br><br>Statistical Package for the Social Science software version 19.0 was used for quantitative analysis and a p-value of <0.05 was considered to be significant.	ACP counseling session tailored towards ACP discussion in the context of dementia and entailed educating patient-caregiver dyads on ACP, exploring their fears and concerns in initiating ACP, and discussions on preferred future care plans, facilitated by a trained dementia nurse who specialises in ACP	d (a & b)	"readiness for ACP" or "willingness"	No control	Pre-post counseling questionnaires assessed the patient-caregiver dyads' perceptions of ACP, and explored their understanding of the purpose of basic ACP and their attitudes and feelings about end-of-life communication with each other, in particular, their understanding of the illness, willingness to have further discussions, and the acknowledgement of barriers to communication. The post-counseling questionnaire also assessed their experience during ACP counseling.	Pre-post counseling: Not mentioned ("We modeled the framework of our questionnaire after a study by Fried, Bradley, Towle, and Allore")	Pre-intervention:<br>(1) 30% of the patients and 50% of the caregivers had heard of ACP;<br>(2) 70% had thought of their healthcare proxy, but only 30% of the patients had made future care plans prior to ACP discussion;<br>(3) 95% of patients surveyed agreed with the importance of communicating to their caregivers about their illnesses;<br>(4) 55% would like further discussion with their caregivers.<br><br>Post-intervention:<br>(1) 68.4% were keen for further discussions despite a reduced proportion who understood the importance of talking about their illness;<br>(2) A higher percentage of caregivers (57.9% post-counseling vs. 45% pre-counseling) acknowledged barriers to communication with a concomitant decline in the number of caregivers (85% to 78.9%) who were keen to explore ACP further;<br>(3) Caregivers were significantly more likely to acknowledge barriers to ACP discussions than patients (57.9% versus 10.5%, respectively; p = 0.005, Fisher's exact test);<br>(4) Caregivers were also more likely to find ACP useful in understanding complications of illness than patients (p = 0.042, Fisher's exact test);<br>(5) More caregivers (78.9% versus 68.4% patients) found ACP helpful in exploring their preferences for future treatment; the difference was not statistically significant (p = 0.714, Fisher's exact test).<br><br>Before ACP counseling, 55% would like further discussion with their caregivers. After ACP counseling, more patients (68.4%) were keen for further discussions. A decline in the number of caregivers (85% to 78.9%) who were keen to explore ACP further.	PwD: a<br>FCG: b	The proportion of caregivers in our study who were keen on further ACP discussions declined although the proportion who agreed to the importance of ACP remained constant after ACP counseling. This contrasted with the patient group, where there was a post-counseling increase in the proportion who were keen to have further ACP discussions despite a reduction in those who deemed this important. The observed post-counseling increase in the proportion who acknowledged barriers to communication in both groups, could be attributable to increased awareness of perceived barriers as a result of ACP counseling. Interestingly, this was associated with a differential response to willingness for further discussion, being increased amongst PWD and decreased among caregivers. The differential perceptions may pose a barrier to continued ACP discussions, given that ACP is a dynamic process that involves engagement of conversations in an iterative manner, reflections, and values clarification.	
37	H.L. Huang; W.R. Lu; C.L. Liu; H.J. Chang	Advance care planning information intervention for persons with mild dementia and their family caregivers: Impact on end-oflife care decision conflicts	PLoS One	2020	Exploring the effects of a family-centered ACP information intervention among persons with dementia and FCGs	(Not mentioned)	Patient-caregiver dyads; Patients with mild dementia and their caregivers	d (a & b)	N=78 (n=40 dyads)	Taiwan	Outpatient clinics of regional teaching hospitals, dementia care centers, or community care centers.	HO + OTH	Pre-post study	Pre-/post-intervention questionnaires	Dyads of persons with mild dementia and their FCGs were recruited from outpatient clinics of regional teaching hospitals, dementia care centers, or community care centers located in Taipei City and New Taipei City, Taiwan.<br><br>Inclusion criteria (patients): (1) > 55 years of age; (2) diagnosed with mild dementia or mild cognitive impairment based on a score of 0.5–1 on the Clinical Dementia Rating (CDR) scale or a score > 18 on the Mini-Mental State Examination (MMSE); (3) had no established ACP; and (4) able to communicate in Mandarin or Taiwanese.<br><br>Inclusion criteria (family caregivers):: (1) > 20 years of age; (2) able to communicate in Mandarin or Taiwanese.<br><br>Both persons with dementia and their primary caregivers had to agree to participate in the study.	No randomization	(Not mentioned)	SPSS 22.0 software for Windows was used for statistical analysis. Descriptive statistics included frequency, percentage, mean, and standard deviation (SD). The effect of the intervention was determined with a paired sample t test and effect size by analyzing the difference in pre-intervention and post-intervention effect scale scores for knowledge of end-stage dementia treatment, the knowledge of and attitude toward ACP, and decisional conflicts for EoL care. A level of p < .05 was considered significant. frequency, percentage, mean, and standard deviation (SD). The effect of the intervention was determined with a paired sample t test and effect size by analyzing the difference in pre-intervention and post-intervention effect scale scores for knowledge of end-stage dementia treatment, the knowledge of and attitude toward ACP, and decisional conflicts for EoL care. A level of p < .05 was considered significant.	The ACP information intervention was conducted in two parts by a senior nurse who was ACP-trained. The first part of the intervention provided persons with dementia and caregivers with an ACP manual, titled, Be My Own Master: Talk about Advance Care Planning for Dementia. The manual included both text as well as pictorial aids, which have been shown to significantly improve understanding and decision-making for persons with mild to moderate dementia. The nurse explained the contents of the manual, which included descriptions about the symptoms of end-stage dementia and the common EoL life-sustaining medical treatments, such as CPR, machine ventilation, tube feeding, intravenous infusion, and antibiotics. The manual also provided details about the benefits and risks of the treatments, as well as the process of establishing ACP and the regulations involved. The second part of the intervention involved family-centered strategies for developing ACP and the nurse facilitated open discussions between the persons with dementia and their FCGs. Dyads were encouraged to communicate their thoughts and expectations about EoL care, and to describe any uncertainties about the process; the nurse intervener provided any needed clarifications. The intervention lasted approximately 60 min.	d (a & b)	"Advance Care Planning Attitude"	No control	Pretest-posttest questionnaires: (a) knowledge of end-stage dementia treatment; (b) knowledge of ACP; (c) Advance Care Planning Attitude (ACPA); (d) Decisional conflict for EoL care.	(a) Knowledge fo end-stage dementia treatment + 9 items about dementia symptoms<br>(b) Self-developed Knowledge of ACP scale<br>(c) Advance Care Planning Attitude (ACPA) scale + 3 items about ACP procedures/payments<br>(d) Modified Decisional Conflict Scale (DCS)	Effects of the ACP information intervention on patients:<br>(1) Scores for knowledge of end-stage dementia treatment and ACP improved significantly (t = −2.79, p = .008, effect size = 0.5; and t = −4.10, p < .001, effect size = 0.6).<br>(2) No significant change was observed in either the positive (33.00 -> 34.98) or negative (23.03 -> 22.93) attitude towards ACP.<br>(3) The mean total score for posttest decisional conflicts in EoL care was significantly lower than pretest for persons with dementia (49.88 ->35.11, t = 4.76, p < .001, effect size = −0.8).<br><br>Effects of the ACP information intervention on family caregivers:<br>(1) Scores for knowledge of end-stage dementia treatment and knowledge of ACP improved significantly (t = −4.77, p < .001, effect size = 0.8; and t = −8.22, p < .001, effect size = 1.2, respectively).<br>(2) A significant decrease in negative scores (24.27 -> 20.65, t = 3.73, p = .001, effect size = −0.7).<br>(3) The total score for decisional conflicts decreased significantly after the intervention (37.34 -> 27.70, t = 3.89, p < .001, effect size = −0.6).	PwD: c<br>FCG: b (no difference in positive attitude) & a (difference in negative attitude)	Effects of intervention on attitude toward ACP:<br>For FCGs, scores for negative attitude significantly decreased. The decline in negative attitude among FCGs following the intervention suggests most caregivers maintained an open attitude during the discussions. In contrast, the persons with dementia may have been less able to discuss their concerns due to age- and disease-related factors. Other studies have shown persons with dementia are less interested in abstract problems unrelated to urgent needs.	
52	Ouchi, K.; Joshi, C.; Kaithamattam, J.; Gale, S.A.; Marshall, G.A.; Pietras, A.; Wang, W.; Boyer, E.W.; Tulsky, J.A.; Block, S.D.; Rentz, D.; Schonberg, M.A.	Refinement of an Emergency Department-Based, Advance Care Planning Intervention for Patients With Cognitive Impairment and Their Caregivers	The Gerontologist	2024	An acceptability study that will inform what population is appropriate for a future randomized control trial (RCT) of ED GOAL.	(Not mentioned)	Patients with established diagnoses of MCI or dementia and their caregivers	d (a & b)	N=26 (dyads)	USA	Tertiary care, academic medical center	HO	Pilot study	(Pre- and) post-intervention questionnaire	we recruited patients with established diagnoses of MCI or dementia and their caregivers from behavioral neurology clinics. For patients with MCI or mild dementia, both the patient and caregiver were the participants of the study. For patients with moderate to advanced dementia, only the caregiver was the participant.<br><br>Inclusion criteria were: (1) aged 50 and older; (2) have established diagnosis of MCI/dementia determined by extensive neurocognitive testings by behavioral neurologists; (3) have the capacity to consent (as noted above, for patients with moderate to advanced dementia, only the caregivers participated in the study, caregiver capacity was not assessed); and (4) English speaking. We excluded patients or caregivers who were determined by the treating clinician to be not fit for this study (e.g., patient lacked insight into dementia).	No randomization	(Not mentioned)	The chi-square test was used to evaluate whether one categorical response statistically predominates within the heard/ understood measures. A one-sample binomial exact test of proportions for categorical outcomes was used to measure baseline and 1-month data for the ACP Engagement Survey.<br>A p value of <.05 as the significance threshold was used. The analysis for this paper was generated using SAS software	ED GOAL is a short, scripted, motivational interview-based intervention, which prompts patients to articulate their values and preferences and communicate them in ACP conversations with their outpatient clinicians and avoids a time-consuming, sensitive conversation in the time-pressured ED environment.	d (a & b)	"ACP engagement" or "ready"	No control	The primary outcome was acceptability of ED GOAL measured immediately after the intervention. Additionally, we examined patient-/caregiver-perceived respectfulness to autonomy as another measure to depict the acceptability.<br>The secondary outcomes included participant-reported ACP engagement before and 1 month after our intervention using the four-item validated measure. In addition, we examined participants' self-reported completion of ACP conversations with their outpatient clinician one month after ED GOAL.	Primary outcomes:<br>(a) Acceptability was defined using a validated measure of "heard and understood" modified to the context of end-of-life care. The validated measure asks, "How much do you feel heard and understood by your doctors, nurses, and health care staff?" We modified this measure to focus on participants' values and preferences toward end-of-life care (i.e., "How much have you felt heard and understood about what you would want in medical care if you were to get sicker?" from 1 = not at all to 5 = completely)<br>(b) Rating of clinician's respectfulness (5 out of 5 Likert scale rating).<br><br>Secondary outcomes:<br>(c) Participant-reported ACP engagement before and 1 month after our intervention using the four-item validated measure.<br>(d) participants' self-reported completion of ACP conversations with their outpatient clinician one month after ED GOAL.	(a) After ED GOALCG, 23% (6/26) of participants reported being completely (5 out of 5 Likert scale rating, median = 3.5, interquartile range [IQR] = 1, p value = .461) heard and understood by their primary outpatient clinicians about what they would want in medical care if they got sicker, while 58% (15/26) of participants reported being completely (median = 5, IQR = 1, p value = .003) heard and understood by their study clinician about what they would want in medical care if they got sicker.<br>(b) Nearly all patients and caregivers (96%, 25/26) reported that the study clinician was very respectful (5 out of 5 Likert scale rating, median = 5, IQR = 0) to elicit what the patient would want in his/her future medical care. Hundred percent (26/26) of caregivers reported that the study clinician was very respectful (median = 5, IQR = 0) to elicit the caregivers' inputs regarding what the patients may want.<br>(c) There was not a statistically significant change in overall ACP engagement as measured by the 4-point validated ACP engagement scale. However, the item "how ready are you to talk to your doctor about the type of care you would like to receive if you were very sick or near the end of life?" increased from 3.4 to 4.2 on the 5-point Likert scale.	a	Our acceptability outcomes suggest that patients living with MCI/dementia and their caregivers found ED GOALCG meaningful and potentially clinically useful.<br><br>In this acceptability study, ED GOALCG did not have a statistically significant impact on ACP behavior, as measured by the ACP Engagement Survey.	
59	Sussman, T.; Lawrence, J.; Pimienta, R.	This is how I want it": Exploring the use of a workbook with persons with dementia to support advance care planning engagement"	Dementia	2022	Exploring the use of one publicly available advance care planning engagement workbook called Your Conversation Starter Kit, exploring the views of persons with dementia and their caregivers on using the workbook to support advance care planning engagement. It further seeks to uncover the conditions that support and hinder workbook use.	(Not mentioned)	Patients with dementia and family members/caregivers	d (a & b)	N=18 (n=10 PWD; n=8 caregivers)	Canada	Alzheimer Society chapters + home care department	OTH + HC	Exploratory sequential mixed methods design	(Phase 1: focus groups)<br><br>Phase 2: pre-intervention questionnaire (users versus non-users)	Purposive sampling methods were used to recruit participants for both study phases. In Phase 1, persons with dementia and their caregivers were recruited from two Alzheimer Society chapters in Canada (one in Quebec and one in Ontario). In Phase 2, participants were recruited from the same Alzheimer Society chapters as well as a home care department in Quebec. In both phases, staff within the recruiting organizations identified potential participants, provided preliminary in formation about the study and, if potential participants were agreeable, passed on relevant contact information to the study research coordinator. All interested potential participants were then contacted by the research coordinator, who reviewed the study's purpose and details of involvement, emphasizing that participation was voluntary and would have no impact on their organization's service provision. Staff recruiting participants in both phases were asked to reach out to potential participants who were (a) French or English speaking (b) clinically judged by staff to be a person with dementia in the early stages of their condition and capable of providing consent to participate in advance care planning discussions, or (c) an informal caregiver supporting a person with dementia capable of participation. Phase 2 recruitment targeted participants who had not participated in Phase 1. Signed written consent was obtained from participating persons with dementia and caregivers in both phases. In Phase 1, written consent was obtained on the day of focus group deliberations. In Phase 2,written consent was obtainedon theday of workbookdistribution. Willingness andcapacity to participate was monitored by members of the research team throughout the duration of the study.	N/a	(Not mentioned)	(Phase 2:) descriptives; means and standard deviations for continuous data and frequencies and percentages for categorical data. We explored differences between users and non-users across overall advance care planning engagement survey scores using an independent 2-tailed t test. All bivariate analyses using overall use/non-use as an outcome measure were conducted using Fishers exact tests.	Your conversation starter kit: The workbook is a publicly available interactive 12-page workbook that is informed by a staged model of advance care planning engagement. The four steps included in the workbook are designed to movepeople through a process of personal reflection and communication. Each section contains open-ended ended questions, while the second section also contains ranked choice questions. The first two steps (Get Ready & Get Set) promote reflection on how to engage with a family member/caregiver in advance care planning discussions through tips, prompts, and questions that focus on values, concerns, and preferences for future care. The last two steps (Go & Keep Going) direct users towards discussions with family/caregivers. The workbook was developed by the Institute for Healthcare Improvement as a part of a public engagement initiative to improve advance care planning awareness and uptake for all adults.	d (a & b)	"Readiness for ACP" and "ACP engagement"	No control	Predictors (based on focus groups):<br>(1) Solo versus dyadic use<br>(2) Prior advance care planning engagement<br><br>Outcome measure: Workbook use:<br>(1) Workbook used or unused<br>(2) Sections of workbook used in-whole or in-part	(1) Solo versus dyadic use: Enrollment (alone versus together)<br>(2) Prior advance care planning engagement: 24-item advance care planning engagement survey; Likert scale ranging from (1), indicating low engagement on a given item, to (5), indicating high engagement.<br><br>(1) Workbook used or unused: workbooks returned with written entries to the interactive elements were considered used. Workbooks returned with no written entries, or not returned at all, were considered unused.<br>(2) Sections of workbook used in-whole or in-part: Interactive elements with written entries were considered used, while interactive elements without written entries were considered unused.	Prior engagement: Workbook users (M=4.33, SD=0.537) were more likely to have engaged in some form of advance care planning prior to workbook use than Non-Users (M=3.40, SD=0.427), t (22)=4.551,p<.01.<br><br>Fourteen of the 24 persons with dementia (58%) enrolled in the study returned completed workbooks. Nine of the 14 workbookusers (64%) used at least one of the two open-ended questions in Step1 and 13 of 14 users (100%) completed at least one of the five open questions in Step 2. Nine of 14 users (64%)used Step 3's one open-ended question while only six of 14 users (43%) completed at least one of the three open-ended questions in Step 4. Recorded responses in Step 4 suggested that those who had reflected on or discussed their wishes with others were satisfied with their engagement.	N/a	Our analysis of workbook distribution revealed that persons with dementia who enrolled with a family member/caregiver were significantly more likely to use the workbook than those enrolling alone. While we cannot speak to how and when persons with dementia engaged their caregivers in workbook use, this finding supports the dyadic approaches to advance care planning engagement endorsed by caregivers during focus group deliberations.<br><br>Our pilot distribution of workbooks intimated that those persons with dementia with prior advance care planning engagement were more likely to use the workbook than those with no prior advance care planning engagement. Hence, while the workbook may be useful for different stages of the advance care planning process, those at the precontemplative stages of advance care planning may be less likely to use it without support or encouragement.	There is no outcome measure of readiness among the quantitative data. The qualitative data does focus more on ACP engagement, so only qualitative data from this study is included in the manuscript.
Expert article 1	Wolff, J.L.; Cagle, J.G.; Hanna, V.; Dy, S.M.; Echavarria, D.; Giovannetti, E.R.; Boyd, C.M.; Saylor, M.A.; Hussain, N.; Reiff, J.S.; Scerpella, D.; Zhang, T.; Sekhon, V.K.; Roth, D.	Sharing health carewishes among older adultswith cognitive impairment in primary care: Results from a randomized controlled trial	Alzheimer's & Dementia	2024	The Sharing Health Care Wishes in Primary Care (SHARE) trial (NCT04593472) was conducted to test the effects of a multicomponent intervention to proactively engage family and normalize ACP among older primary care patients with cognitive impairment, ranging from mild to severe.	Our hypothesis was that the intervention protocol would lead to better quality of communication about end-of-life care with primary care clinicians (primary outcome) and ACP processes (secondary outcomes) at 6 months.	Patients with mild,, moderate and severe cognitive impairment and family caregivers (care partners)	d (a & b)	N=273 dyads	USA	Primary care practices	GP	single-blind randomized controlled trial	pre- and post-intervention interviews (6- and 12-month follow-up)	Patients of participating clinicians were mailed letters describing the study 1 month in advance of a scheduled visit. Patients who did not "opt out" by mail were contacted by the research team to discuss study procedures and administer a telephone screening interview. For patients who were unable to interact by telephone, eligibility was assessed through completing the survey with a knowledgeable informant. For those who expressed interest care partners of eligible patients were contacted to introduce the study and administer a telephone screening interview. Upon obtaining informed consent, research staff scheduled telephone or video enrollment meetings to conduct baseline assessments with patients and care partners.<br><br>Eligible patients were ≥80 years and screened positive for cognitive impairment (1+ incorrect answer or unable to respond to a six-item screening survey).16 Eligible care partners were 18 years or older, English-speaking, did not report a life-threatening illness, and did not screen positive for cognitive impairment. Exclusion criteria included plans to move out of state within the year.	Randomizationwas at the dyad level. After obtaining informed consent and completing baseline interviews, each dyad was randomized to the experimental or control condition in a 1:1 ratio using stratified, blocked randomization with randomly varying block sizes of four or six dyads for each clinician. Allocation concealment was ensured by blinding the principal investigator and staff conducting follow-up assessments and responsible for coding study outcomes.	(Not mentioned)	We used intention-to-treat analyses as the primary method of analysis. Analyses of covariance (ANCOVAs) were used to evaluate treatment effects on continuous outcomes (quality of communication about endof-life care and readiness to engage in ACP), with treatment group as the primary independent variable and baseline score as a key covariate. For dichotomous outcomes, we constructed logistic regression models. Baseline measures were examined and considered for inclusion as covariates in adjusted models if imbalance was observed by treatment group at baseline or when correlated with outcomes at follow-up after adjusting for baseline score. As severity of patient cognitive impairment (mild vs moderate/severe, following standard TICS-m cut points) was imbalanced by treatment group at baseline, this measure was included as a covariate in adjusted analyses. Thus, adjusted analyses control for baseline instrument scores (for continuous measures) and severity of patient cognitive impairment.We examined the consistency of intervention effects for subgroups identified a priori by relationship (spouse vs non-spouse) and by cognitive impairment (mild vs moderate or severe). All analyseswere conducted using SAS version 9.4with Proc GLM used for the ANCOVA and Proc LOGISTIC used for the logistic regression analyses.	The Sharing Health Care Wishes in Primary Care (SHARE): Usual care + (1) Letter from primary care practice introducing a new initiative to improve communication with information about agenda setting, the patient portal and advance care planning; (2) ongoing access to a trained facilitator to lead advance care planning discussions; (3) patient-family agenda-setting to discuss the role of family and stimulate discussion about goals of care and advance care planning; (4) facilitated registration to patient's electronic health record (patient portal) for patient and family.	d (a & b)	"readiness to engage in ACP"	Participants assigned to the control group received minimally enhanced usual care, encompassing an introductory letter, the guide for communicating with clinicians,18 and a blank advance directive. Control dyads were told that they were participating in a study about communication in primary care.	Patient readiness to engage in ACP	four-item ACP engagement survey, with each item measured on a five-point scale (range: 0 to 20).24,25 Care partner readiness to engage in ACP draws on parallel items for surrogate decision-makers,26 supplemented by two items specific to the surrogate decision-maker perspective (range: 0 to 30), as previously described.12	Intervention care partners reported somewhat greater readiness to engage in ACP than control care partners at baseline (24.58 and 23.00; p = .051). After adjusting for baseline score and severity of cognitive impairment, care partner readiness to engage in ACP was greater in the intervention than control group at 6 months (27.01 and 24.82; p = .010), but this effect was not sustained at 12 months (25.96 and 25.19; p = .435). Patient readiness to engage in ACP was greater in the intervention than control group at baseline (16.29 and 14.41; p = .014). In adjusted models, we did not observe a significant treatment effect at 6months (17.25 vs 16.21; adj p = .459) but readiness to engage in ACP was greater among intervention than control patients at 12 months (18.00 vs 15.52; p = .042).	a	Intervention care partners and patients reported greater readiness to engage in ACP at 6 and 12 months, respectively, and were more likely to report having completed selected key aspects of ACP. Care partners – and especially non-spouses – were more likely to report having discussed patient preferences about end-of-life care, while patients were more likely to report having named a surrogate decision-maker.<br><br>Observed increases in patient- and care partner-reported readiness to engage in ACP and aspects of ACP completion provide evidence that it is possible to facilitate productive conversations while balancing patient and care partner perspectives that do not always align.35	
Expert article 2	Yeang, C.C.; Ho, K.H.; Chan, Y.L.	A dyadic advance care planning intervention for people with early-stage dementia and their family caregivers in a community care setting: a feasibility trial	BMC Geriatrics	2023	This study was a feasibility trial, the results of which were used to inform a subsequent definitive pragmatic randomised controlled trial. The primary objectives of this feasibility trial were as follows:<br>1. To assess the feasibility of intervention procedures and of subject recruitment in terms of the recruitment rate;<br>2. To assess the feasibility of subject retention in terms of the attrition rate at all time points;<br>3. To identify participants' reasons for refusal to participate and dropout throughout the study period; and<br>4. To assess the acceptability of the intervention to participants with early-stage dementia and their family caregivers in terms of the completion rate and satisfaction score.<br><br>(The secondary objectives of this feasibility trial were as follows:<br>1. To assess the average duration required to deliver the intervention in a real community care setting;<br>2. To assess the feasibility of the study instruments in measuring the study outcomes in terms of data completeness for a subsequent definitive trial; and<br>3. To estimate the potential effects of the intervention.)	(Not mentioned)	People with dementia & family caregivers	d (a & b)	N=36 dyads	China	elderly community centres	OTH	Experimental, pretest–posttest study	Pre- and post-intervention questionnaires (& post-intervention interviews)	The staff members of each participating centre reviewed their client database, identified the potential participants, and introduced the study to them through phone or face to face contact. Those who were interested in the study were referred to the research team. The first author then screened the eligibility of the potential participants in person and explained the study details to those who were eligible to participate. Written consent to participate was sought from eligible participants with early-stage dementia and their family caregivers.<br><br>Eligible participants were Cantonese-speaking Chinese adults who i) were aged 55 years or above, ii) were at stage 3 or 4 on the Global Deterioration Scale, and iii) had a designated family member willing to participate in this study. People with dementia were assumed with full mental capacity according to recommendations for ACP in dementia unless they were unable to communicate and comprehend adequately the information for informed consent process during subject recruitment. They were excluded if they i) had undergone ACP, ii) had previously signed an advance directive, or iii) had a terminal illness with a life expectancy of less than 6 months. The inclusion criteria for the designated family caregiver were as follows: they were i) nominated by the participants with early-stage dementia and were involved in making care- and medical-related decisions for him/her, ii) aged 18 years or above, and iii) able to speak Cantonese. Paid caregivers were excluded.	(No randomization)	(Not mentioned)	Quantitative data were analysed using the IBM SPSS Statistics version 26.0. Descriptive statistics were used to present the sociodemographic and clinical characteristics of the participants, recruitment rate, intervention completion rate, participant satisfaction scores, attrition rate, and missing rate for study instruments at various time points. The homogeneity of the participants in the two study groups was examined by comparing their characteristics using Chi-square test for categorical variables and the Mann–Whitney U test for continuous variables. Any significant group differences were identified as covariates and adjusted in the outcome analysis. Generalised estimating equations (GEE) were employed to examine the differential changes in study outcomes at T1, T2 and T3 with respect to T0. Effect size was measured by dividing the mean difference between two groups by the pooled standard deviation and reported as Cohen'd value. The cut-off points of small, medium and large effect size measured by Cohen'd are 0.2, 0.5 and 0.8, respectively. All analysis was considered significant at p value less than 0.05 (2-tailed).	Have a Say (HAS) programme: a culturally appropriate dyadic ACP intervention for Chinese people with early-stage dementia, underpinned by the Bandura's self-efficacy model. It ncompasses four sources of self-efficacy in its components: (1) Vicarious experience: participants learn the concept and benefits of ACP by watching micro-movies on the life-stories of two persons with dementia, one who has completed ACP and one who has not; (2) Verbal persuasion: participants receive verbal information about the dementia trajectory, future care needs, surrogate role in decision-making and advance directives, and encouragement for participating in ACP discussions; (3) Emotional arousal: participants are supported by ACP facilitators to express their feelings and concerns throughout the ACP process; and (4) Performance accomplishment: participants are actively engaged and guided by the ACP facilitators to express their care preferences.<br><br>Week 1: Format: (1) A nurse-led group-based session; (2) Micro-movie and video watching; (3) Group discussion. Objectives: (1) Increase participants' understanding of dementia and advance care planning; (2) Support participants to understand and share their views about future health decline; (3) Increase participants' understanding of future care options; (4) Raise participants' awareness of ACP. Content: (1) Introduce the nature and trajectory of dementia and the care needs of people with dementia; (2) Share the life stories of two persons with dementia, one who has completed ACP and one who has not, through micro-movie; (3) Introduce information about ACP, including its rationale, contents, and benefits, using video clips; (4) Introduce various life-sustaining treatment options using video clips and demonstrate medical equipment, e.g. endotracheal tube and feeding tube; (5) Facilitate group discussion among participants to share their views about future health decline, end-of-life care preferences, and surrogate decisionmaking<br>Week 2: Format: (1) ACP facilitator-led dyadic sessions; (2) Individualised ACP discussion guided by an ACP booklet. Objectives: (1) Explore participants' values, preferences, and family resources and dyads' concerns related to future care planning; (2) Provide information on various options for each future care issue; (3) Facilitate the discussion.<br>Content: Discussion topics covered: Personal values, wishes, and views about own health and future care, Place of care, Daily care arrangements, Feeding options.<br>Week 3: Objectives: (1) Support the dyads to make decisions about future care options; (2) Document participants' views and preferences in the ACP booklet. Content: Discussion topics covered: Life-sustaining treatments at the end-of-life stage, Legal and financial arrangements, Surrogate decision-makers, Funeral arrangement, Other unfulfilled wishes.	d (a & b)	"Readiness for ACP (engagement)"	Dyads in the attention control group received three sessions consisting of educational health talks related to ageing that were neither dementia-specific nor related to ACP. The sessions, with each lasted approximately 1 h, were held on a weekly basis to equalize the contact time and attention given to the participants in the intervention group.<br><br>The participating elderly community centres were pairmatched based on their scope of service and scale. The two centres from each pair were assigned to the intervention group or the control group at a 1:1 ratio by an independent research assistant. Only the superintendents of the participating centres were notified about the group allocation results; the staff members who assisted in subject recruitment and a research assistant who conducted outcome assessments were blinded to group allocation.	Self-efficacy and readiness for ACP engagement	ACP Engagement Survey. The 9-item version comprised two subscales, one each measuring selfefficacy and readiness for ACP engagement. Its Chinese version has been validated with sound psychometric properties. Each item is scored from 1 to 5, with a higher total score representing a higher level of ACP engagement. Those who did not respond according to the response format and chose the "uncertain" or "refuse" response, was counted as invalid response. During data analysis, these responses were replaced by "1" which indicated "not confident at all" in the self-efficacy subscale or "I have not thought about this before" in the readiness subscale.	The participants with dementia in the intervention group reported a significantly greater improvement, with large effect sizes, in self-efficacy for ACP at T3 (p = 0.030; d = 0.98) and readiness for ACP at T1 (p = 0.006; d = 1.23), compared with those in the control group.<br><br>BUT: The participants were able to complete all study instruments except the ACP engagement survey. The proportion of invalid responses among participants with dementia in the self-efficacy subscale of the ACP engagement survey at T0, T1, T2, and T3 were 13.9%, 21%, 19.8%, and 26.4% respectively, and that in the readiness subscale were 1.9%, 0%, 10.9%, and 10.9%, respectively.	a	The findings suggested that the HAS programme has the potential to enhance the participants' readiness for ACP immediately after the intervention although the effects did not sustain,	
Expert article 5	Dupont C; Smets T; Monnet F; Pivodic L; De Vleminck A; Audenhove C; Van den Block L	A website to support people with dementia and their family caregivers in advance care planning: Results of a mixed-method evaluation study	Patient Education and Counseling	2024	The aims of this study are:<br>(1. To evaluate the usability, acceptability and feasibility of the ACP support website;)<br>(2. To explore the experiences of people with dementia and their family caregivers with the use of the website;)<br>3. To evaluate the effects of using the website on people with dementia and family caregivers' ACP readiness, knowledge, attitudes, perceived barriers to engage in ACP, self-efficacy, and skills to engage in ACP.	(Not mentioned)	N=52 (n=21 PwD; n=31 FCG)	People with dementia (mild to moderate) and family caregivers	d (a & b)	Belgium	Organisations involved in dementia (care) (community?) & memory clinics	HO + OTH	Mixed-methods (convergent parallel mixed-methods design)	(Pre- and post-intervention interviews and) questionnaires + ecological momentary assessment (evaluation during intervention)	• For both people with dementia and their family caregivers:<br>Participants were recruited via organisations involved in dementia (care) and neurologists working in memory clinics. We asked the (health) professionals to identify and approach potential participants who met the eligibility criteria and might be interested and eligible for this study, ensuring that the recruitment organisations first confirmed the dementia diagnosis of potential participants. When potential participants were interested in the study, they were asked to contact or wanted to meet the researcher directly when s/he was present in the hospital. Potential participants who contacted the researcher received an information letter and a consent form. If the person was still interested in participating after receiving the study information, the researcher made an appointment with the participant(s) to do the eligibility screening and ask for informed consent (i.e. onboarding meeting).<br><br>Eligibility criteria:<br>• Having an interest in and being willing to test the ACP website<br>• Being able to consent to study participation<br>• Speaking and understanding Dutch<br>• Having a device (laptop, tablet, mobile phone, etc.)<br>• Did not participate in the cognitive testing of study materials<br>• For people with dementia: is diagnosed with mild to moderate young- or late-onset dementia<br>• For the family caregiver: takes active care (physical, emotional, social, etc.) of the person with dementia<br>• For participants recruited as dyads: At least one of the participants needs to be able to navigate the website (e.g. the person with dementia and the family caregiver cannot both have visual impairments or other disabilities that prevent them from interacting with the ACP website)	(No randomization)	(Not mentioned)	Descriptive statistics were used to analyze ACP engagement	The website contains:<br>• Information about ACP and its legal frameworks, why ACP is important, and reasons to discuss ACP with the healthcare profession is supported by videos. The website allows users to navigate according to their preferences, allowing a non-linear approach, i.e., people with dementia and their families can use the website based on their needs and ACP readiness levels.<br>• Support for communication by tips and videos on how to start talking about ACP and how to introduce the topic with the healthcare professional.<br>• Information about the documentation of ACP and referral to healthcare professionals<br>• Two interactive communication support tools to support reflection and communication about what matters most and social aspects of future care and treatment, end-of-life preferences: a card game with preformulated statements based on the GoWish cards developed in the US and a fill-in tool with open-end questions about "what matters to you".<br>• Several functionalities to support accessibility of the information, e.g. text-to-speech, enlarge-the-text and contrast options (appendix 1 and 2 for screenshots of the website and the interactive tools and summary overview of all pages).	d (a & b)	"Readiness for ACP (engagement)"	No control	ACP readiness	4-item ACP engagement Survey (Dutch version)	The proportion of persons with dementia who indicated in the ACP readiness survey that they wanted or discussed possibilities with family about future care and treatment increased from 57 % at baseline to 67 % after the study period. For family caregivers, this increased from 68 % to 77 %	a	This evaluation study observed increased ACP readiness	
66	Vellani S; Puts M; Iaboni A; McGilton KS	Voice your values, a tailored advance care planning intervention in persons living with mild dementia: A pilot study	Palliative and Supportive Care	2022	In this paper, we report on the following specific objectives:<br>1. Examine the feasibility of implementing the VYV intervention in community-dwelling older adults with early-stage dementia as measured by recruitment and retention rates and intervention fidelity.<br>2. Identify the influence of the VYV intervention on trusted individuals' level of decision-making confidence, dementia knowledge, and psychological distress when compared to their baseline levels.<br>3. Determine the effect of the VYV on PLwD's level of engagement in the ACP process and psychological distress when compared to their baseline levels.	(Not mentioned)	N=40 (n=20 PwD; n=20 FCG)	People with mild dementia and family caregivers	d (a & b)	Canada	Outpatient geriatric clinics	OTH	Pretest–posttest study	Pre- and post-intervention questionnaires	Participants were English-speaking community-dwelling individuals of 65 years and older who had a diagnosis of mild dementia of any type and confirmed by the referring physician or a nurse practitioner. There was no restriction on the time since the diagnosis as long as the individuals were in the mild stage at the time of their participation in the study. Participants had access to a device with an internet connection for videoconferencing and a trusted individual with at least weekly contact who could enroll in the study. PLwD were not eligible if they had an acute psychotic disorder, and/or clinical depression, due to concerns that these conditions could limit their ability to participate meaningfully in ACP discussions; blindness or deafness; or if they already had a written advance care plan in place. A written advance care plan was any document that identifies a person's values, wishes, and/or future goals of care. Living wills, power of attorney, and financial planning documents were not considered ACP documents. English-speaking trusted individuals 18 years and older were included, who did not have dementia, blindness, or deafness, and had access to a device for videoconferencing.	(No randomization)	(Not mentioned)	Paired sample t-test was conducted to compare the mean score on all measures before and after the VYV intervention and the change in outcome measures (difference, 95% confidence interval). Preliminary effect sizes of changes were calculated. The McNemar test was used to identify the percentage of participants in the precontemplative phase of behavior change at baseline and the percentage of those who moved forward in the behavior change using the readiness subscale of the ACP Engagement Survey.	The VYV intervention: An intervention based on empirical data and the theoretical underpinnings of the Representational Approach to Patient Education and the Transtheoretical Model of Stages of Change. The process of delivering the intervention was guided by a selection of recommendations by Piers and colleagues: (1) timing to initiate ACP, (2) capacity evaluation, (3) carrying out ACP discussions, (4) role of those close to the person with dementia, and (5) documentation of wishes. The intervention consisted of two sessions using videoconferencing: (1) The first session consisted of gathering information about the participant's understanding of dementia, the desire to learn about dementia and ACP, life goals, fears and worries related to the dementia diagnosis. (2) The second session consisted of a summary from the first session, tailored education about dementia and ACP, addressing in-the-moment questions by the participants, coaching the participants to identify vales and wishes for future healthcare (related to terminal and vegetative states), and documentation of values and wishes.	d (a & b)	"Readiness" and "ACP engagement"	No control	ACP readiness	15-item ACP engagement survey	There was a significant difference in the mean scores of PLwD on the ACP engagement survey. 55% (n = 11) of the PLwD were in the pre-contemplative stage of behavior change at T1, which decreased to 40% (n = 8) post-intervention, showing upward movement in the behavior change stage, though it was not statistically significant (p = 0.32). [no report of FCG readiness]	PwD: a	This evaluation study observed increased ACP readiness	
