# Supplementary material for: Communication strategies for improving readiness for advance care planning in dementia: A systematic review
Source: J Alzheimers Dis. 2026 Jun 26;112(4):1735–50. doi: 10.1177/13872877261461216 (PMC13433894; doi:10.1177/13872877261461216)
Supplement: Supplementary file 3 [file sj-pdf-4-alz-10.1177_13872877261461216.pdf]

Supplemental Table 5. Qualitative data extraction sheets of included studies

									a=Patients b=Caregivers c=Healthcare staff d=Mix			GP=General practice HC=Home care HO=Hospital NH=Nursing home OTH=Other								a=Patient level b=Caregiver level c=Healthcare staff d=Mix			
Study information							Participants						Study methods								Outcomes		Reviewer comments
#	Authors	Title	Journal	Year	Research aim	Research question(s)	Participants	Participants	N	Country	Setting	Setting	Design	Methods of data collection	Recruitment/sampling procedures (+inclusion/exclusion criteria)	Qualitative methodology	Phenomena of interest	Readiness definition	Intervention description (if applicable)	Intervention target (if applicable)	Themes/categories	Author conclusions	
1	N, Ali, P, Anthony, W, S, Lim, M, S, Chong, E, W, H, Poon, V, Drury, M, Chan	Exploring Differential Perceptions and Barriers to Advance Care Planning in Dementia among Asian Patient–Caregiver Dyads–A Mixed-Methods Study	Int J Environ Res Public Health	2021	(1) Examine the disparity in perceptions among subjects with mild dementia and their caregivers towards ACP after undergoing ACP counseling and (2) Explore the barriers to ACP in dementia	(Not mentioned)	Patient-caregiver dyads; Patients with mild dementia and their caregivers	d (a & b)	n = 40 (20 dyads)	Singapore	Memory clinic	HO	Parallel mixed-method	Post-intervention interviews (and pre-/post-intervention questionnaires)	Recruitment from outpatient attendees of the Memory Clinic at a tertiary hospital over a period of 2.5 years. Inclusion criteria: (1) Newly diagnosed with dementia based on the Diagnostic Statistical Manual IV criteria; (2) Mild severity, as defined by a global Clinical Dementia Rating score (CDR) of 0.5–1; (3) No significant mood disorders or behavioral problems; (4) Had an identified caregiver who was conversant in English. Exclusion criteria: (1) Moderate or severe dementia (CDR ≥ 2); (2) Who had a concordant diagnosis of delirium; (3) Those with unstable medical conditions, defined as more than 2 hospitalizations in the last 1 month prior to recruitment.	Thematic analysis	(1) To enable the subjects and caregivers to elaborate further on their perceptions and feelings with regards to ACP, and (2) to identify barriers to ACP discussion.	"readiness for ACP"	The dementia nurse (P.A.), a trained ACP facilitator, scheduled an ACP counseling session with the patients and their respective caregivers after they had been apprised of the dementia diagnosis. The content of the session was tailored towards ACP discussion in the context of dementia and entailed educating patient–caregiver dyads on ACP, exploring their fears and concerns in initiating ACP, and discussions on preferred future care plans. The session was conducted in the form of a semi-structured interview and usually lasted for an hour. A short debriefing session was arranged to address any concerns or queries that might have surfaced during the counseling session and follow-up plans were carried out if required.	d (a & b)	(1) Patient-related factors (transference of decision making, poor cognition and lack of understanding, and dis-inclination to plan for the future);<br>(2) caregiver-related factors (perceived negative impact on the patient, caregiver discomfort, and confidence in congruent decision making);<br>(3) socio-cultural factors (taboos, superstitions, and religious beliefs);<br>(4) the inappropriate timing of discussions.	In a collectivist Asian culture, socio-cultural factors pose important barriers, and a family-centric approach to initiation of ACP may be the first step towards engagement in the ACP process.<br>(1) Patient-Related Barriers: (a) The transference of decision making to other family members may be viewed as the proper social order to maintain the familism and conduct of family duties, and a showcase of filial piety in Asian culture. This inevitably propagates the proclivity of not planning for the future should the patient's health status deteriorate to the point of incapacity. (b) The cognitive deficits experienced by PWD may preclude them from fully participating in ACP conversations due to the lack of understanding of what ACP entails and lack of appreciation of the future end-of-life care, as reported in this study. (c) People with cognitive impairment can still participate meaningfully in ACP conversations, with the listener being vigilant to discern their preferences and values.<br>(2) Caregiver-Related Barriers: (a) Caregivers were also more likely to find ACP useful in understanding the complications of illness, but they revealed their discomfort in broaching end-of-life issues due to the perceived negative impact of such discussions on patients; (b) A unique finding in our study was the perceived congruence in future care decisions by the caregivers. This may help explain the reluctance among caregivers to engage in further ACP discussion post-counseling. Such perceptions may result in avoidance behavior to engage in ACP as caregivers attempt to navigate through existential tensions confronted during discussions on end-of-life care as dementia progresses to the terminal stage in their loved ones.<br>(3) Collectivist Culture Influence Patient–Caregiver Dyads' Perception of ACP and Socio-Cultural Barriers to ACP: Consistent with the esteemed values of the Asian culture, the acceptance of decision making by the caregiver (usually adult children) on behalf of the infirmed parent is an unspoken responsibility akin to an act of filial piety. There seems to be an implicit understanding that caregivers will know the preferences and wishes of PWD though no prior discussions had occurred. (b) In a collectivist society with pervasive cultural differences, such as Singapore, culture has a direct influence on communication. Topics such as death, end of life, and sickness are not explicitly or frequently discussed; thus, it is important to maintain sensitivity towards such topics when discussed. Our study highlighted the contribution of cultural diversity as a communication barrier to ACP, such as the twin taboo of dementia and ACP; superstitious beliefs surrounding ACP discussions; and spiritual and religious beliefs. A person's culture shapes the way they think and how they make meaning out of illness, suffering, and dying. Hence, culture inevitably plays an important role in influencing decision making towards the end of life.<br>(4) Timing of ACP Initiation and Discussion: (a) Patient–caregiver dyads viewed good health and early stage of dementia as indicators to delay ACP discussions. This is tantamount to a retrograde shift from the contemplation/discussion phase to the pre-contemplation phase. ACP discussions can only be initiated when patients and caregivers are ready to be engaged in such sensitive dialogues, hence emphasizing the need to tailor such discussions to individual readiness and openness. Nevertheless, beginning ACP early in the course of dementia can provide opportunities for ongoing and gradual conversations to slowly engage patients and caregivers in participating in the planning process. (b) A possible complex array of factors need to be considered to determine the optimal timing of ACP discussion in dementia, such as acceptance of the diagnosis of dementia, understanding of the trajectory of illness and future cognitive decline, decision-making capacity, readiness to engage in emotionally laden conversations, and the ability to decide on preferences based on future clinical scenarios.	The qualitative data does not provide information regarding communication strategies influencing readiness; it focuses on barriers for ACP and generally emphasizes that readiness is crucial. Qualitative data from this study is not described in the manuscript.
38	Sussman, T., Lawrence, J., Pimienta, R.	"This is how I want it": Exploring the use of a workbook with persons with dementia to support advance care planning engagement"	Dementia	2022	Exploring the use of one publicly available advance care planning engagement workbook called Your Conversation Starter Kit, exploring the views of persons with dementia and their caregivers on using the workbook to support advance care planning engagement. It further seeks to uncover the conditions that support and hinder workbook use.	(Not mentioned)	Patients with dementia and family members/caregivers	d (a & b)	N=18 (n=10 PWD; n=8 caregivers)	Canada	Alzheimer Society chapters	OTH + HC	Exploratory sequential mixed-methods design	Phase 1: focus groups<br><br>(Phase 2: pre-intervention questionnaire (users versus non-users))	Purposive sampling methods were used to recruit participants for both study phases. In Phase 1, persons with dementia and their caregivers were recruited from two Alzheimer Society chapters in Canada (one in Quebec and one in Ontario). In Phase 2, participants were recruited from the same Alzheimer Society chapters as well as a home care department in Quebec. In both phases, staff within the recruiting organizations identified potential participants, provided preliminary information about the study and, if potential participants were agreeable, passed on relevant contact information to the study research coordinator. All interested potential participants were then contacted by the research coordinator, who reviewed the study's purpose and details of involvement, emphasizing that participation was voluntary and would have no impact on their organization's service provision. Staff recruiting participants in both phases were asked to reach out to potential participants who were (a) French or English speaking (b) clinically judged by staff to be a person with dementia in the early stages of their condition and capable of providing consent to participate in advance care planning discussions, or (c) an informal caregiver supporting a person with dementia capable of participation. Phase 2 recruitment targeted participants who had not participated in Phase 1. Signed written consent was obtained from participating persons with dementia and caregivers in both phases. In Phase 1, written consent was obtained on the day of focus group deliberations. In Phase 2 written consent was obtained on the day of workbook distribution. Willingness and capacity to participate was monitored by members of the research team throughout the duration of the study.	Four-step semantic thematic analysis	Acceptability of the workbook: (1) perceived benefits of using the workbook, (2) perceived challenges of using the workbook, (3) reactions to the workbook content, and (4) recommendations for workbook implementation	"Readiness for ACP" and "ACP engagement"	Your conversation starter kit: The workbook is a publicly available interactive 12-page workbook that is informed by a staged model of advance care planning engagement. The four steps included in the workbook are designed to move people through a process of personal reflection and communication. Each section contains open-ended ended questions, while the second section also contains ranked choice questions. The first two steps (Get Ready & Get Set) promote reflection on how to engage with a family member/caregiver in advance care planning discussions through tips, prompts, and questions that focus on values, concerns, and preferences for future care. The last two steps (Go & Keep Going) direct users towards discussions with family/caregivers. The workbook was developed by the Institute for Healthcare Improvement as a part of a public engagement initiative to improve advance care planning awareness and uptake for all adults.	d (a & b)	(1) The workbook can be used alone or with others<br>(2) Workbook supports initial and ongoing ACP engagement<br>(3) Interactive elements and probes about decisional involvement viewed as useful<br><br>"Although many participants agreed that conversations about future care can be intimidating, comments across groups suggested that the workbook had potential to initiate and support advance care planning engagement. Participants who had no prior engagement with advance care planning felt the workbook could be used to "open the conversation" because the content "gives ideas" and 'good prompts' for what to think about and discuss. The direction offered by the workbook was viewed as useful for initial advance care planning engagement because people "don't always know what to [think about] because[they]have not faced it yet". Participants who had prior experience with advance care planning also saw utility in the workbook because they felt it could reinforce, maintain, or elaborate on former future care discussions and reflections."	Regarding the timing of advance care planning engagement, our focus groups deliberations suggested that persons with varying levels of prior advance care planning engagement saw value in the workbook. For those who had not engaged in any advance care planning contemplation, the workbook was seen as an opportunity to express what they may want to have happen in the event their health declines. For those who had engaged in prior reflections or discussions, the workbook was seen as an opportunity to revisit prior conversations and clarify preferences and wishes.	
52	Van Rickstal, R., De Vleminck, A., Aldridge, M.D., Van der Steen, J.T., Engelborghs, S., Van den Block, L.	Limited engagement in, yet clear preferences for advance care planning in young-onset dementia: An exploratory interview-study with family caregivers	Palliative Medicine	2019	To explore: (1) whether family caregivers had already engaged in advance care planning with patients and/or professionals and the accompanying reasons and (2) family caregivers' preferences for how to ideally engage in the process with patients, family and professionals.	(Not mentioned)	Family caregivers	b	N=15	Belgium	(1) a day care centre (De Toren); (2) a volunteer organization and (3) the Department of Neurology's Memory Clinic at Hospital Network of Antwerp.	OTH (2) + HO	Qualitative (Not specified)	Semi-structured interviews	Participants were recruited from three Flemish sites: (1) a day care centre (De Toren); (2) a volunteer organization (Het Ventiel), both specifically aimed at people with young-onset dementia and their families and (3) the Department of Neurology's Memory Clinic at Hospital Network of Antwerp. Coordinators at each site approached eligible subjects, asking for participation and for permission to give their contact information to the researchers. Participants were contacted by phone or email by R.V.R. or A.D.V., address ing any questions and ascertaining willingness to participate. After agreement, interviews were scheduled at a mutually convenient time, at a place of respondents' choice. No potential respondent contacted by the researchers refused participation.<br><br>Eligible respondents needed to be (1) the main family caregiver of a person formally diagnosed with young-onset dementia (regardless of dementia sub type), (2) aged 18 or older, (3) Dutch-speaking and (4) needed to have given written informed consent to participate.	Thematic analysis?	(1) the extent to which patients, family and/or professional caregivers had already engaged in advance care planning and the reasons for doing so or not and (2) their preferences for how to ideally engage in advance care planning with the patient, family and/or professional caregiver(s).	"Engagement in ACP"	n/a	n/a	(1) Extent of engagement in planning for the future and accompanying reasons<br>A1) The current conception of advance care planning: A1.1 Plans perceived by caregivers as elements of advance care planning; A1.2 Euthanasia; A1.3 The completion of advance directives.<br>A2) Reasons for not discussing advance care planning: A2.1 Not considering advance care planning significant or useful; A2.2 Patient behaviour hindering discussions; A2.3 Adopting a day-to-day attitude; A2.4 Caregivers' need for self-protection; A2.5 Questioning patients' cognitive capacity for advance care planning.<br>(2) Preferences for advance care planning in young onset dementia<br>B1) Who, when and what?: B1.1 Who?; B1.2 When?; B1.3 What?<br>B2) The need for information as a first step towards advance care planning: B2.1 About disease trajectory; B2.2 About advance care planning; B2.3 About services.<br>B3) The need for high-quality care.<br>B1.1: "All respondents who shared thoughts on the ideal person to initiate advance care planning named a third party and mainly physicians, because for relatives themselves, it could be hard to assume this role. ... However, physicians' attitudes also played a role: respondents distinguished between honest and empathic physicians versus those who didn't seem to see the patient as a person. One person felt a peer, or a 'buddy', would be best suited to introduce advance care planning to persons with young-onset dementia."<br>B1.2: The ideal timing for initiating advance care planning was perceived slightly differently by several caregivers. The majority felt that initiation should take place timely. ... However, some also noted the necessity of allowing the patient time to process the diagnosis. ... Other recommendations entailed establishing a trusting relationship between patient and physician prior to advance care planning and repeatedly discussing preferences instead of just once."<br>B1.3: "Some caregivers indicated that advance directives could be a means to start a broader conversation about future care to emphasize patients' remaining abilities, rather than emphasize their acquired limitations. Finally, several mentioned an 'individual approach' is of great importance."<br>B2.1: Five respondents explicitly stated they – including the patient – were not or barely given any information about what to expect regarding disease progression and its implications, sometimes despite their explicit request. For the few that did receive this type of information, the majority added they did so only after specifically asking for it themselves. Most caregivers argued that uncertainty and unpredictability of an individual disease trajectory makes it very difficult for physicians to provide concrete information. In addition, 'you can't just scatter all of people's hope at once'. Indeed, one caregiver stated too much information 'would only make her depressed'.<br>B2.2: Many respondents were unfamiliar with the term advance care planning or its purpose, with only a few being informed about it by their physician. Several respondents expressed the need to let people know 'advance care planning exists' and the need to 'explain advance care planning in a simple manner'.<br>B2.3: Unravelling information about services for persons with young-onset dementia was a major challenge for caregivers (and patients). Almost all respondents reported there is no clear overview of available services: they recommended that care options in young-onset dementia are clarified, centralized and made easily accessible.<br>B3: The emphasis placed on a person-centred approach became evident from the recommended 'individual approach' for advance care planning, but was also expressed regarding the need for appropriate care services for people with young-onset dementia, accustomed to their age, activity level and clinical presentation. However, this was rarely the case."	"This suggests that it is paramount to adequately inform patients and caregivers about (general) expectations so they can prepare themselves if they wish so. ... Despite information on advance care planning, these poor services might cause or amplify the perception of advance care planning as pointless: engaging in the process would not meaningfully contribute to the care received since this care is flawed anyway."<br><br>"As the interviewed family caregivers in our study pointed out, care for persons with dementia at a younger age should take into account their distinct age, higher activity level and the specific clinical presentation of their condition. Part of this recommendation could be interpreted as yet another example of the strong protective role that family caregivers of persons with young-onset dementia take on: appropriate care tailored to younger patients with dementia should focus on their remaining abilities, and these might be found in other domains, for instance in physical possibilities, as compared to older persons with dementia."<br><br>"Our study highlights the importance of availability of high-quality care in the context of advance care planning in young-onset dementia, as quality of care<br>might form a strong incentive to (meaningfully) plan for it and might substantially counter the perception of advance care planning as useless for people with young onset dementia."	
Expert article 3	Abshire Saylor, M.A., Hanna, V., Zheng, P., Thai, Q.H., Green, C.M., Cagle, J.G., Wolff, J.L.	Advance care planning in adults ages 80 years and older with impaired cognition: Using actual conversations to examine best practices	Alzheimer's & Dementia	2024	(First, we sought to quantitatively characterize how ACP conversation duration and verbal activity of older adult, family care partner, and ACP facilitators differed among those with normal cognition, those with mild cognitive impairment, and those suspected of having dementia.)<br>Second, we sought to qualitatively explore the nature of older adult and care partner conversations separately by older adults' level of cognitive impairment, while focusing on the three issues identified by the international consensus relating to capacity, family, and engagement and communication.	(Not mentioned)	N=88 dyads	Older adults with cognitive impairment & family caregivers (care partners)	d (a & b)	USA	Primary care practices	GP	Qualitative (Not specified)	Facilitated ACP discussions (transcript analysis)<br><br>This is a secondary analysis of data collected from the intervention arm of the SHARE trial. We draw on a unique dataset of 92 recorded ACP conversations involving 88 older adults with cognitive impairment (average age = 88 years), family care partners, and trained ACP facilitators.	Briefly, participants included persons who screened positive for having cognitive impairment and were 80 years or older, spoke English, were able to provide informed consent themselves or through their proxy decisionmaker, and regularly attended primary care visits with an eligible family care partner who was the person they identified as "helping the most" when making decisions about their health. Participants were recruited from eight primary care practices in the Baltimore–Washington, DC, area, and after providing written informed consent and completing a baseline survey, were randomized 1:1 to the intervention or control group. Institutional Review Board approval was received from Johns Hopkins University. Written informed consent was obtained from patients and care partners prior to data collection.	Thematic analysis	Three issues identified by the international consensus relating to capacity, family, and engagement and communication.	"Engagement in ACP"	(SEE EXPERT ARTICLE 1) The SHARE intervention included four components: (1) a letter from the practice introducing the initiative to prepare persons and families to discuss goals of care, (2) access to a trained facilitator to lead ACP discussions, (3) person-family agenda setting to align perspectives about the role of family, and (4) facilitated registration to the patient portal (for patient and family) to extend electronic interactions and information access to family.	d (a & b)	THEME 3: ENGAGEMENT AND COMMUNICATION – Structured questions about values and strong ACP communication strategies elicit engagement from older adults with dementia. … We identified examples of successful strategies to increase older adult participation with the involvement of family when needed. Subthemes include that (1) values elicitation engages older adults and (2)ACP communication strategies elicit older adult approval of care, a form of engagement.<br>Theme 3.1 Values elicitation to engage older adults<br>The Respecting Choices conversation guide begins by clarifying the purpose ofACPand asking about what constitutes "a good day" or good quality of life. These structured elements were successful in engaging older adults across all levels of cognitive impairment, including those with dementia.<br>ACP Facilitator: If you were having a good day, what would happen on that day? Who would you talk to? What would you do? What does living well mean to you? Care Partner: What would you consider a good day? Do you consider what we do here every day nothing? Patient: I'd sit there, watch TV or walk out to the trashcans and walk out for a little bit, pick up the mail and stuff like that. (Values-elicitation questions regarding cultural and spiritual values resonated with some older adults by allowing them to share information about their faith and the communities with whom they identified (ie veterans, ethnic community). In one case, having an ACP facilitator with cultural congruence to the family and older adult helped elicit engagement and communication of the older adultwhose TICS-m score was consistent with dementia. ACP Facilitator: Yeah, so I know being from the islands, I also love being outdoors, so I understand. Patient: We're not used to be – Yeah, because we were growing up, we have a front door and a back door . . . You know? And here, you're in the apartment. It's one door out.No other doors to go . . . Mm-hmm. Culture. ACP Facilitator: All the island people are just so friendly. Patient: Very. Very friendly . . . Whether they have money or not, they're friendly.)<br>Theme 3.2 ACP communication strategies elicit older adult approval of care, a form of engagement<br>ACP facilitators successfully used communication strategies such as repetition and mirroring to confirm their understanding of older adult values. This was particularly important when care partners said more than patients during the ACP conversation. These communication strategies allowed older adults with dementia to affirm their wishes. ACP Facilitator: I think what I'm appreciating out of our conversation so far is there's great communication within the family with [patient] and the children and that way. . . Patient:Oh, absolutely. ACP Facilitator: Yes, ma'am. And [care partner], accepting this role as the primary healthcare agent, when youwere selected, were there any fears or concerns or reservations about the possibility of having to make decisions for Mom in case she can't speak for herself? Care Partner: No, because we've been close enough – I've known her long enough, of course. So, she lets her feelings known. Patient:Mywishes.	Values elicitation and ACP communication techniques (ie, repetition and mirroring) were identified as effective strategies for eliciting the participation and engagement of older adults, including those with dementia.	
Expert article 5	Dupont C., Smets T., Monnet F., Pivodic L., De Vleminck A., Audenhove C., Van den Block L.	A website to support people with dementia and their family caregivers in advance care planning: Results of a mixed-method evaluation study	Patient Education and Counseling	2024	The aims of this study are: (1). To evaluate the usability, acceptability and feasibility of the ACP support website.)<br>2. To explore the experiences of people with dementia and their family caregivers with the use of the website.<br>(3. To evaluate the effects of using the website on people with dementia and family caregivers' ACP readiness, knowledge, attitudes, perceived barriers to engage in ACP, self-efficacy, and skills to engage in ACP.)	(Not mentioned)	N=52 (n=21 PwD; n=31 FCG)	People with dementia (mild to moderate) and family caregivers	d (a & b)	Belgium	Organisations involved in dementia (care) (community?) & memory clinics	HO + OTH	Mixed-methods (convergent parallel mixed-methods design)	Pre- and post-intervention interviews (and questionnaires + ecological momentary assessment)	• For both people with dementia and their family caregivers:<br>Participants were recruited via organisations involved in dementia (care) and neurologists working in memory clinics. We asked the (health) professionals to identify and approach potential participants who met the eligibility criteria and might be interested and eligible for this study, ensuring that the recruitment organisations first confirmed the dementia diagnosis of potential participants. When potential participants were interested in the study, they were asked to contact or wanted to meet the researcher directly when s/he was present in the hospital. Potential participants who contacted the researcher received an information letter and a consent form. If the person was still interested in participating after receiving the study information, the researcher made an appointment with the participant(s) to do the eligibility screening and ask for informed consent (i.e. onboarding meeting).<br><br>Eligibility criteria:<br>• Having an interest in and being willing to test the ACP website<br>• Being able to consent to study participation<br>• Speaking and understanding Dutch<br>• Having a device (laptop, tablet, mobile phone, etc.)<br>• Did not participate in the cognitive testing of study materials<br>• For people with dementia: is diagnosed with mild to moderate young- or late-onset dementia<br>• For the family caregiver: takes active care (physical, emotional, social, etc.) of the person with dementia<br>• For participants recruited as dyads: At least one of the participants needs to be able to navigate the website (e.g. the person with dementia and the family caregiver cannot both have visual impairments or other disabilities that prevent them from interacting with the ACP website)	Thematic analysis	Experiences of people with dementia and their family caregivers with the use of the website	"Readiness for ACP (engagement)"	The website contains:<br>• Information about ACP and its legal frameworks, why ACP is important, and reasons to discuss ACP with the healthcare professional is supported by videos. The website allows users to navigate according to their preferences, allowing a non-linear approach, i.e., people with dementia and their families can use the website based on their needs and ACP readiness levels.<br>• Support for communication by tips and videos on how to start talking about ACP and how to introduce the topic with the healthcare professional.<br>• Information about the documentation of ACP and referral to healthcare professionals<br>• Two interactive communication support tools to support reflection and communication about what matters most and social aspects of future care and treatment, end-of-life preferences: a card game with preformulated statements based on the GoWish cards developed in the US and a fill-in tool with open-end questions about "what matters to you".<br>• Several functionalities to support accessibility of the information, e.g. text-to-speech, enlarge-the-text and contrast options (appendix 1 and 2 for screenshots of the website and the interactive tools and summary overview of all pages).	d (a & b)	(1) ACP knowledge: At baseline, some participants (n = 12) mentioned in the interviews they did not know what ACP was, but most participants (n = 40) indicated they had some knowledge about ACP. Participants (n = 25) often mentioned euthanasia and documentation as the main topic of ACP. Thirteen of these participants mentioned that, concerning ACP, "everything was taken care of". However, when asked what was "taken care of", these participants mainly mentioned financial affairs (n = 13). After the study, most participants (n = 31) said they better understood ACP even though they indicated they already knew the topic, while others (n = 16) mentioned their knowledge did not change much because of their prior knowledge.<br>(2) Attitudes towards the website and towards ACP: At baseline, all participants had positive attitudes torwards ACP, with some participants mentioning during the interviews that they did not yet think about or discuss preferences for future care or treatment (n = 26) or wishes around the end of life (n = 31). Only a few wanted to<br>or had previously discussed ACP with their healthcare professional (n = 11). In the follow-up interviews, all participants expressed again positive attitudes towards using the website and engaging in ACP and thought the website may be helpful to facilitate the ACP process. After the eight weeks more people (n = 36) said they discussed preferences for future care and treatment or wishes around the end of life (n = 24). Also, after eight weeks, more people discussed or wanted to discuss ACP with their healthcare professionals (n = 21). However, after using the website, five family caregivers felt it was too late to discuss ACP with the person with dementia because the person with dementia was unable, according to them, to think or discuss ACP because of the moderate stage of dementia. They felt ACP was important, especially to talk about what is important and had some regrets about not starting earlier.<br>(3) Perceived barriers to engage in ACP: At baseline, the most mentioned barrier to engage in ACP expressed by people with dementia (n = 15) during the interviews was the desire to live in the present and consider themselves too healthy to think about ACP. Family caregivers (n = 23) often mentioned the business of everyday life as a reason to not start with ACP. Family caregivers (n = 15) also found it challenging to anticipate the changes associated with dementia and needed support to start the conversation. After the eight weeks, participants mentioned the same barriers, but particularly, family caregivers felt the ACP website could support overcoming some of these barriers. The interactive communication tools and testimonial videos on the website were valued as helpful in this area. Family caregivers underlined they found the tip "talk together, rather than solely focusing on the person with dementia" useful. However, people with dementia (n = 3) and family caregivers (n = 10) perceived anticipating and formulating wishes in advance as challenging because of the uncertainties associated with dementia. Three family caregivers and three people with dementia still perceived the same barriers as they did at baseline and did not see how to overcome them.<br>(4) Self-efficacy to use the website and to start ACP: At baseline, all family caregivers and 17 people with dementia believed they could use the website independently without assistance. In the interviews, over half of the participants said they had initiated ACP together before the start of the study, what aligns with what participants indicated in the ACP readiness survey. In follow-up interviews, all family caregivers reported they used the website alone, while six people with dementia required assistance from their partners. Overall, after the eight weeks, 34 participants indicated that they used the website to start thinking about potential future care preferences. Some of them (n = 14) made an appointment with their physician to discuss these thoughts and/or indicated they (n = 16) downloaded or got an advance directive, which they wanted to discuss with family and a healthcare professional. All participants who used the website would recommend it to others, highlighting they felt it could benefit others. Moreover, those who started thinking and talking about ACP during the eight weeks through the website expressed a desire to continue using it, including reconsidering past discussions, and exploring additional ACP topics they were not ready to discuss, like nursing home placement.<br>(5) Skills to engage in ACP: At baseline, some participants (n = 27) indicated they did not knowhow to start thinking and discussing ACP – besides filling in advance directives. Others felt they could find their way if they searched the internet or would ask their healthcare professional (n = 25). When participants had already discussed or documented ACP before their participation in this study, a healthcare professional (n = 10) or a notary (n = 20) was involved in most cases. After the eight weeks study period, all participants indicated in the interview that the website could help them to develop skills to reflect on and have conversations about what is important for them and potential future care preferences, for example, by using communication tips or interactive communication tools. Family caregivers of dyads indicated they first used the website alone – to learn what ACP is and how to start discussing the future – and then involved the person with dementia. This way, twenty-eight participants discussed potential future care needs and what they would or would not want. For example, some dyads used the interactive communication card tool to start a conversation about potential future care and care possibilities. For instance, in one couple, the cards "dying at home" and "being surrounded by family and friends" triggered a conversation about future care and potential placement in a nursing home.	This evaluation study showed that people with dementia and family caregivers considered the website useful and valued the ACP content. They felt that the website supported them in several part of ACP. The website improved participants' ACP knowledge, self-efficacy, skills and reported fewer barriers to engaging in ACP after 8 weeks compared to the beginning of the study. Participant attitudes toward ACP remained positive throughout the study. Participants expressed a desire to<br>continue using the website and would recommend using it to others. However, people with dementia found the website less user-friendly than family caregivers, and family caregivers mostly used the website alone or with the person with dementia, with only three people with dementia indicating they used the website alone.	The qualitative data does not provide information regarding communication strategies influencing readiness; it focuses on ACP knowledge, attiutudes towards the intervention, perceived barriers for ACP, self-efficacy to use the intervention, and skills to engage in ACP. Qualitative data from this study is not described in the manuscript.
