# Supplementary material for: Communication strategies for improving readiness for advance care planning in dementia: A systematic review
Source: J Alzheimers Dis. 2026 Jun 26;112(4):1735–50. doi: 10.1177/13872877261461216 (PMC13433894; doi:10.1177/13872877261461216)
Supplement: Supplementary file 4 [file sj-docx-1-alz-10.1177_13872877261461216.docx]

**Supplemental Material**

**Communication strategies for improving readiness for advance care planning in dementia: A systematic review**

**Supplemental Table 1.** Search terms for each electronic database searched

| Electronic database | Search term |
| --- | --- |
| PubMed | ("Dementia"[Mesh] OR "Dementia*"[tiab] OR "Alzheimer* disease"[Mesh] OR "Alzheimer*"[tiab] OR "AD"[tiab] OR "ATD"[tiab] OR "Mild cognitive impairment"[tiab] OR "MCI"[tiab] OR "Major neurocognitive disorder"[tiab] OR "Major NCD"[tiab] OR "Vascular dementia"[tiab] OR "VaD"[tiab] OR "Lewy body dementia"[tiab] OR "LBD"[tiab] OR "Lewy Body Disease"[Mesh] OR "Lewy Body"[tiab] OR "Lewy bodies"[tiab] OR "Dementia with Lewy bodies"[tiab] OR "DLB"[tiab] OR "Creutzfeldt-Jakob Syndrome"[Mesh] OR "Creutzfeldt-Jakob Disease"[tiab] OR "CJD"[tiab] OR "Parkinson’s Disease dementia"[tiab] OR "Wernicke-Korsakoff syndrome"[tiab] OR "Frontal lobe dementia"[tiab] OR "Frontotemporal dementia"[tiab] OR "FTD"[tiab] OR "Frontotemporal dementia"[Mesh] OR "Temporal lobe dementia"[tiab] OR "Mixed dementias"[Mesh] OR "Mixed dementia*"[tiab] OR "Cognitive dysfunction"[Mesh] OR "Cognitive dysfunction*"[tiab] OR "Neurocognitive disorders"[Mesh] OR "Neurocognitive disorder*" [tiab] OR "Korsakoff Syndrome"[Mesh] OR "Korsakoff Syndrome"[tiab] OR "Wernicke Encephalopathy"[Mesh] OR "Wernicke Encephalopathy"[tiab] OR "cognitive disorder*"[tiab] OR "Glioma"[Mesh] OR "Glioma"[tiab]) AND ("Readiness"[tiab] OR "Ready"[tiab] OR "Willingness"[tiab] OR "Receptiveness"[tiab] OR "Capability"[tiab] OR "Acceptability"[tiab] OR "Preparedness"[tiab] OR "Capacity"[tiab] OR "Aptness"[tiab] OR "Engagement"[tiab] OR "Stage* of Change"[tiab] OR "Behavior change"[tiab] OR "Patient acceptance of Health care"[Mesh] OR "Patient involvement"[tiab] OR "Patient participation"[tiab] OR "Patient empowerment"[tiab] OR "Patient activation"[tiab]) AND ("Discussion tool"[tiab] OR "Communication aid"[tiab] OR "Conversation guide"[tiab] OR "Conversation tool"[tiab] OR "Conversation starter"[tiab] OR "interviewing"[tiab] OR "interview"[tiab] OR "Communication"[Mesh] OR "Communication strateg*"[tiab] OR "Communication technique*"[tiab] OR "Communication approach"[tiab] OR "Conversation strateg*"[tiab] OR "Conversation technique*"[tiab] OR "Conversation approach"[tiab] OR "Health Communication"[Mesh] OR "Health Communication"[tiab] OR "Teach-Back Communication"[Mesh] OR "Teach-Back communication"[tiab] OR "Teach back communication"[tiab] OR "Guiding tools"[tiab] OR "Shared decision making"[tiab]) AND ("Advance Care planning"[tiab] OR "Advance Care planning"[Mesh] OR ACP[tiab] OR "Proactive care planning"[tiab] OR "End-of-life planning"[tiab] OR "End-of-life care planning"[tiab] OR "End-of-life decision-making"[tiab] OR "Personalized care planning"[tiab] OR "Future care planning"[tiab] OR "Advance directive"[tiab] OR "Advance healthcare directive"[tiab] OR "Advance medical directive"[tiab] OR "Future care planning"[tiab] OR "Living will"[tiab]) |
| The Cochrane Library | ((Dementia* OR "Alzheimer* NEXT disease" OR "Alzheimer*" OR AD OR ATD OR "Mild cognitive impairment" OR MCI OR "Major neurocognitive disorder" OR "Major NCD" OR "Vascular dementia" OR VaD OR "Lewy body dementia" OR LBD OR "Lewy Body Disease" OR "Lewy Body" OR "Lewy bodies" OR "Dementia with Lewy bodies" OR DLB OR "Creutzfeldt-Jakob Syndrome" OR "Creutzfeldt-Jakob Disease" OR CJD OR "Parkinson’s Disease dementia" OR "Wernicke-Korsakoff syndrome" OR "Frontal lobe dementia" OR "Frontotemporal dementia" OR FTD OR "Temporal lobe dementia" OR "Mixed NEXT dementia*" OR "Cognitive NEXT dysfunction*" OR "Neurocognitive NEXT disorders*" OR "Korsakoff Syndrome" OR "Wernicke Encephalopathy" OR "Cognitive NEXT disorder*" OR Glioma):ti,ab,kw) AND ((Readiness OR Ready OR Willingness OR Receptiveness OR Capability OR Acceptability OR Preparedness OR Capacity OR Aptness OR Engagement OR "Stage* of Change" OR "Behavior change" OR "Patient acceptance of Health care" OR "Patient involvement" OR "Patient participation" OR "Patient empowerment" OR "Patient activation"):ti,ab,kw) AND (("Discussion tool" OR "Communication aid" OR "Conversation guide" OR "Conversation tool" OR "Conversation starter" OR interviewing OR interview OR Communication OR "Communication NEXT strateg*" OR "Communication NEXT technique*" OR "Communication approach" OR "Conversation NEXT strateg*" OR "Conversation NEXT technique*" OR "Conversation approach" OR "Health Communication" OR "Health Communication" OR "Teach-Back Communication" OR "Teach-Back communication" OR "Teach back communication" OR "Guiding tools" OR "Shared decision making"):ti,ab,kw) AND (("Advance Care planning" OR "Proactive care planning" OR "End-of-life planning" OR "End-of-life care planning" OR "End-of-life decision-making" OR "Personalized care planning" OR "Future care planning" OR "Advance directive" OR "Advance healthcare directive" OR "Advance medical directive" OR "Future care planning" OR "Living will"):ti,ab,kw) |
| Embase (Ovid) | ((Dementia* OR "Alzheimer* disease" OR "Alzheimer*" OR AD OR ATD OR "Mild cognitive impairment" OR MCI OR "Major neurocognitive disorder" OR "Major NCD" OR "Vascular dementia" OR VaD OR "Lewy body dementia" OR LBD OR "Lewy Body Disease" OR "Lewy Body" OR "Lewy bodies" OR "Dementia with Lewy bodies" OR DLB OR "Creutzfeldt-Jakob Syndrome" OR "Creutzfeldt-Jakob Disease" OR CJD OR "Parkinson’s Disease dementia" OR "Wernicke-Korsakoff syndrome" OR "Frontal lobe dementia" OR "Frontotemporal dementia" OR FTD OR "Temporal lobe dementia" OR "Mixed dementia*" OR "Cognitive dysfunction*" OR "Neurocognitive disorder*" OR "Korsakoff Syndrome" OR "Wernicke Encephalopathy" OR "Cognitive disorder*" OR Glioma).ab,ti) AND ((Readiness OR Ready OR Willingness OR Receptiveness OR Capability OR Acceptability OR Preparedness OR Capacity OR Aptness OR Engagement OR "Stage* of Change" OR "Behavior change" OR "Patient acceptance of Health care" OR "Patient involvement" OR "Patient participation" OR "Patient empowerment" OR "Patient activation").ab,ti.) AND (("Discussion tool" OR "Communication aid" OR "Conversation guide" OR "Conversation tool" OR "Conversation starter" OR interviewing OR interview OR Communication OR "Communication strateg*" OR "Communication technique*" OR "Communication approach" OR "Conversation strateg*" OR "Conversation technique*" OR "Conversation approach" OR "Health Communication" OR "Health Communication" OR "Teach-Back Communication" OR "Teach-Back communication" OR "Teach back communication" OR "Guiding tools" OR "Shared decision making").ab,ti.) AND (("Advance Care planning" OR "Proactive care planning" OR "End-of-life planning" OR "End-of-life care planning" OR "End-of-life decision-making" OR "Personalized care planning" OR "Future care planning" OR "Advance directive" OR "Advance healthcare directive" OR "Advance medical directive" OR "Future care planning" OR "Living will").ab,ti.) |
| APA PsycINFO | (TX (Dementia OR "Alzheimer* disease" OR Alzheimer* OR "Mild cognitive impairment" OR "Major neurocognitive disorder" OR "Vascular dementia" OR "Lewy body dementia" OR "Lewy Body Disease" OR "Lewy Body" OR "Lewy bodies" OR "Dementia with Lewy bodies" OR "Creutzfeldt-Jakob Syndrome" OR "Creutzfeldt-Jakob Disease" OR "Parkinson’s Disease dementia" OR "Wernicke-Korsakoff syndrome" OR "Frontal lobe dementia" OR "Frontotemporal dementia" OR FTD OR "Temporal lobe dementia" OR "Mixed dementia*" OR "Cognitive dysfunction*" OR "Neurocognitive disorder*" OR "Korsakoff Syndrome" OR "Wernicke Encephalopathy" OR "Cognitive disorder*" OR Glioma)) AND (TX (Readiness OR Read* OR Willingness OR Receptiveness OR Capability OR Acceptability OR Preparedness OR Capacity OR Aptness OR Engagement OR "Stage* of Change" OR "Behavior change" OR "Patient acceptance of Health care" OR "Patient involvement" OR "Patient participation" OR "Patient empowerment" OR "Patient activation")) AND (TX ("Discussion tool" OR "Communication aid" OR "Conversation guide" OR "Conversation tool" OR "Conversation starter" OR Interviewing OR Interview OR Communication OR "Communication strateg*" OR "Communication technique*" OR "Communication approach" OR "Conversation strateg*" OR "Conversation technique*" OR "Conversation approach" OR "Health Communication" OR "Health Communication" OR "Teach-back communication" OR "Teach back communication" OR "Guiding tools" OR "Shared decision making")) AND (TX ("Advance care planning" OR ACP OR "Proactive care planning" OR "End-of-life planning" OR "End-of-life care planning" OR "End-of-life decision-making" OR "Personalized care planning" OR "Future care planning" OR "Advance directive" OR "Advance healthcare directive" OR "Advance medical directive" OR "Future care planning" OR "Living will")) |
| CINAHL | (TX (Dementia OR "Alzheimer* disease" OR Alzheimer* OR AD OR ATD OR "Mild cognitive impairment" OR MCI OR "Major neurocognitive disorder" OR "Major NCD" OR "Vascular dementia" OR VaD OR "Lewy body dementia" OR LBD OR "Lewy Body Disease" OR "Lewy Body" OR "Lewy bodies" OR "Dementia with Lewy bodies" OR DLB OR "Creutzfeldt-Jakob Syndrome" OR "Creutzfeldt-Jakob disease" OR CJD OR "Parkinson’s Disease dementia" OR "Wernicke-Korsakoff syndrome" OR "Frontal lobe dementia" OR "Frontotemporal dementia" OR FTD OR "Frontotemporal Dementia" OR "Temporal lobe dementia" OR "Mixed dementias" OR "Mixed dementia*" OR "Cognitive dysfunction" OR "Cognitive dysfunction*" OR "Neurocognitive disorders" OR "Neurocognitive disorder*" OR "Korsakoff Syndrome" OR "Wernicke Encephalopathy" OR "Cognitive disorder*" OR Glioma)) AND (TX (Readiness OR Read* OR Willingness OR Receptiveness OR Capability OR Acceptability OR Preparedness OR Capacity OR Aptness OR Engagement OR "Stage* of Change" OR "Behavior change" OR "Patient acceptance of Health care" OR "Patient involvement" OR "Patient participation" OR "Patient empowerment" OR "Patient activation")) AND (TX ("Discussion tool" OR "Communication aid" OR "Conversation guide" OR "Conversation tool" OR "Conversation starter" OR Interviewing OR Interview OR Communication OR "Communication strateg*" OR "Communication technique*" OR "Communication approach" OR "Conversation strateg*" OR "Conversation technique*" OR "Conversation approach" OR "Health Communication" OR "Health Communication" OR "Teach-back communication" OR "Teach back communication" OR "Guiding tools" OR "Shared decision making")) AND (TX ("Advance care planning" OR ACP OR "Proactive care planning" OR "End-of-life planning" OR "End-of-life care planning" OR "End-of-life decision-making" OR "Personalized care planning" OR "Future care planning" OR "Advance directive" OR "Advance healthcare directive" OR "Advance medical directive" OR "Future care planning" OR "Living will")) |

**Supplemental Table 2.** Quality appraisal of all included studies using the Mixed Methods Appraisal Tool (MMAT) version 2018

| **Study** | **Design** | **Criterion 1** | **Criterion 2** | **Criterion 3** | **Criterion 4** | **Criterion 5** | **Overall quality** |
| --- | --- | --- | --- | --- | --- | --- | --- |
| **Huang et al. (2020) [45]** | Quantitative (descriptive) | Sampling strategy relevant  **Yes** | Representative sample  **Yes** | Appropriateness of measurements  **Yes** | Low risk of nonresponse bias  **Yes** | Appropriateness of statistical analysis  **Yes** | 5/5 |
| **Ali et al. (2021) [44]** | Mixed-methods | Rational for mixed-methods  **Yes** | Integration of components  No | Interpretation of integration  **Yes** | Addressing inconsistencies  **Yes** | Quality criteria adherence  **Yes** | 4/5  Rationale:  The quantitative and qualitative data were not fully integrated. |
| **Sussman et al. (2022) [47]** | Mixed-methods | Rational for mixed-methods  **Yes** | Integration of components  No | Interpretation of integration  **Yes** | Addressing inconsistencies  **Yes** | Quality criteria adherence  **Yes** | 4/5  Rationale:  The quantitative and qualitative data were not fully integrated. |
| **Vellani et al. (2022) [53]** | Quantitative (non-randomized) | Representativeness of participants  **No** | Appropriateness of measurements  **Yes** | Completeness of outcome data  **Yes** | Accounting for confounders  **Yes** | Administration of intervention as intended  **Yes** | 4/5  Rationale:  The retention rate of participants was low. |
| **Yeung et al. (2023) [52]** | Quantitative (non-randomized) | Representativeness of participants  **No** | Appropriateness of measurements  **Yes** | Completeness of outcome data  **No** | Accounting for confounders  **No** | Administration of intervention as intended  **Yes** | 2/5  Rationale:   (1) There are some differences between the intervention group and control group: living situation (more in intervention group who live alone) and stage of dementia (more in intervention group with higher deterioration score); (2) The authors describe the engagement survey was not completed; (3) Confounding factors were not taken into account. |
| **Ouchi et al. (2024) [46]** | Quantitative (descriptive) | Sampling strategy relevant  **Yes** | Representative sample  **No** | Appropriateness of measurements  **Yes** | Low risk of nonresponse bias  **No** | Appropriateness of statistical analysis  **Yes** | 3/5  Rationale:   (1) There is low inclusion of patients with mild cognitive impairment and the authors reported non-representativeness of sample; (2) There is a high risk of nonresponse bias due to high attrition of participants without clear reason (i.e., no show or not able to be reached). |
| **Wolff et al. (2024) [51]** | Quantitative (randomized controlled trial) | Randomization appropriately performed  **Yes** | Comparability of groups at baseline  No | Completeness of outcome data  **Yes** | Blinding of outcome assessors  **Yes** | Adherence to intervention of participants  **Yes** | 4/5  Rationale: Dementia severity varied between the control group and the intervention group at baseline. |
| **Dupont et al. (2024) [50]** | Mixed-methods | Rational for mixed-methods  **Yes** | Integration of components  **Yes** | Interpretation of integration  **Yes** | Addressing inconsistencies  **Yes** | Quality criteria adherence  **Yes** | 5/5 |
| **Van Rickstal et al. (2019) [48]** | Qualitative | Appropriateness of qualitative approach **Yes** | Adequateness of data collection methods  **Yes** | Findings derived from data  **Yes** | Interpretation substantiated  **Yes** | Coherence between sources, collection analysis and interpretation  **Yes** | 5/5 |
| **Abshire Saylor et al.**^40^ | Qualitative | Appropriateness of qualitative approach **Yes** | Adequateness of data collection methods  **Yes** | Findings derived from data  **Yes** | Interpretation substantiated  **Yes** | Coherence between sources, collection analysis and interpretation  **Yes** | 5/5 |

**Supplemental Table 3.** List of full-text screening of articles. (See PDF).

**Supplemental Table 4.** Quantitative data extraction sheets of included studies (See PDF).

**Supplemental Table 5.** Qualitative data extraction sheets of included studies (See PDF)
